# Manure nutrient recovery, removal, and reuse on California dairies

**DOI:** 10.1002/jeq2.70292

**Published:** 2026-10-22

**Authors:** Stephen R. Kaffka, Cole Smith, Elias Marvinney

**Affiliations:** ^1^ Department of Plant Sciences University of California Davis California USA; ^2^ Department of Land, Air, and Water Resources University of California Davis California USA; ^3^ Department of Civil and Environmental Engineering University of California Davis California USA

## Abstract

California is the leading US dairy state, with over 1.7 million cows. Surplus nutrients in manure, especially nitrogen (N), present significant environmental challenges to local air and water quality. Novel nutrient recovery technologies can reduce adverse manure nitrogen impacts, including losses to groundwater, while increasing the availability of recycled nitrogen, phosphorus, and organic amendments for nondairy farms, creating a circular nutrient economy. We estimated and characterized surplus N on dairy farms in high‐priority N management zones in California's San Joaquin Valley by location and farm size (537 farms). Stocking rates and correlated nutrient surpluses were not a function of overall farm size in terms of either on‐site land available for manure application or mature cow numbers. Using geospatial, techno‐economic, and life cycle assessment modeling, we compared environmental performance (impacts and benefits) of three prominent systems for managing dairy N surplus: vermifiltration, chemical flocculation, and ammonia stripping and recovery. Based on modeled greenhouse gas and nutrient flows within and from dairy manure management systems, tradeoffs between climate impacts and nutrient surplus reduction were observed among these technologies. The largest combined climate and surplus N management benefits occurred when nutrient recovery was combined with renewable natural gas recovery from anaerobic digesters. The collocation of dairies in some subregions affects potential solutions to managing surplus manure N and other nutrients.

## Plain Language Summary

Groundwater nitrate‐N pollution occurs in areas of California with large numbers of dairy farms. Variation in surplus N among dairy farms located in priority nitrogen management zones in the San Joaquin Valley was characterized based on data reported to the regional Water Quality Control Board—estimated using average values for N conserved in solid and liquid manure, differences in animal housing (free stall and open lot dairies) and related manure management systems, estimated crop N uptake for typical cropping systems based on cereal and corn silage crops, and reported stocking rates (SRs). Surpluses accumulated yearly on farms with ≥8–9 mature cows plus replacements per ha and increased with SR in liquid and solid manure. Farm size (total cow numbers) was not correlated with N surpluses. Surplus N must be exported to avoid GW pollution or treated with systems for denitrification or recovery and removal. Selected treatment systems were analyzed for greenhouse gas emissions.

**Abbreviations**
AMMP: Alternative Manure Management Program
AR: application ratio
CV: Central Valley
CVDRMP: Central Valley Dairy Representative Monitoring Program
DAF: dissolved air flotation
DM: dry matter
GHGs: greenhouse gases
LCA: life cycle assessment
LM: liquid manure
MVR: mechanical vapor recompression
NMZ: nitrogen management zone
PMN: potential mineralizable N
SJV: San Joaquin Valley
SM: solid manure
SPARCS‐LCA: scalable, process‐based, agronomic‐responsive cropping system life cycle assessment model
SR: stocking rate
SSJV: Southern San Joaquin Valley
VERM: vermifiltration

## 1 INTRODUCTION

Dairy operations generate large amounts of nutrient‐rich manure, typically used as fertilizer for on‐site feed crop production. California is home to the largest dairy herd in the United States, and milk production is the leading agricultural commodity in the state (Matthews & Sumner, 2019; Njuki, 2022). Assessing and remediating adverse effects of dairy manure management on groundwater nitrate pollution are important California state priorities (CV Salts, 2025). Currently, there is limited understanding of the geographic distribution and scale of dairy operations on N surpluses, as well as clear policy recommendations for farm‐level N recovery technologies. This paper provides a spatial perspective on farm‐level manure N surpluses while evaluating the potential for selected novel manure N recovery technologies to produce surplus N for use on nondairy cropland in California (Figure 1).

**FIGURE 1:**
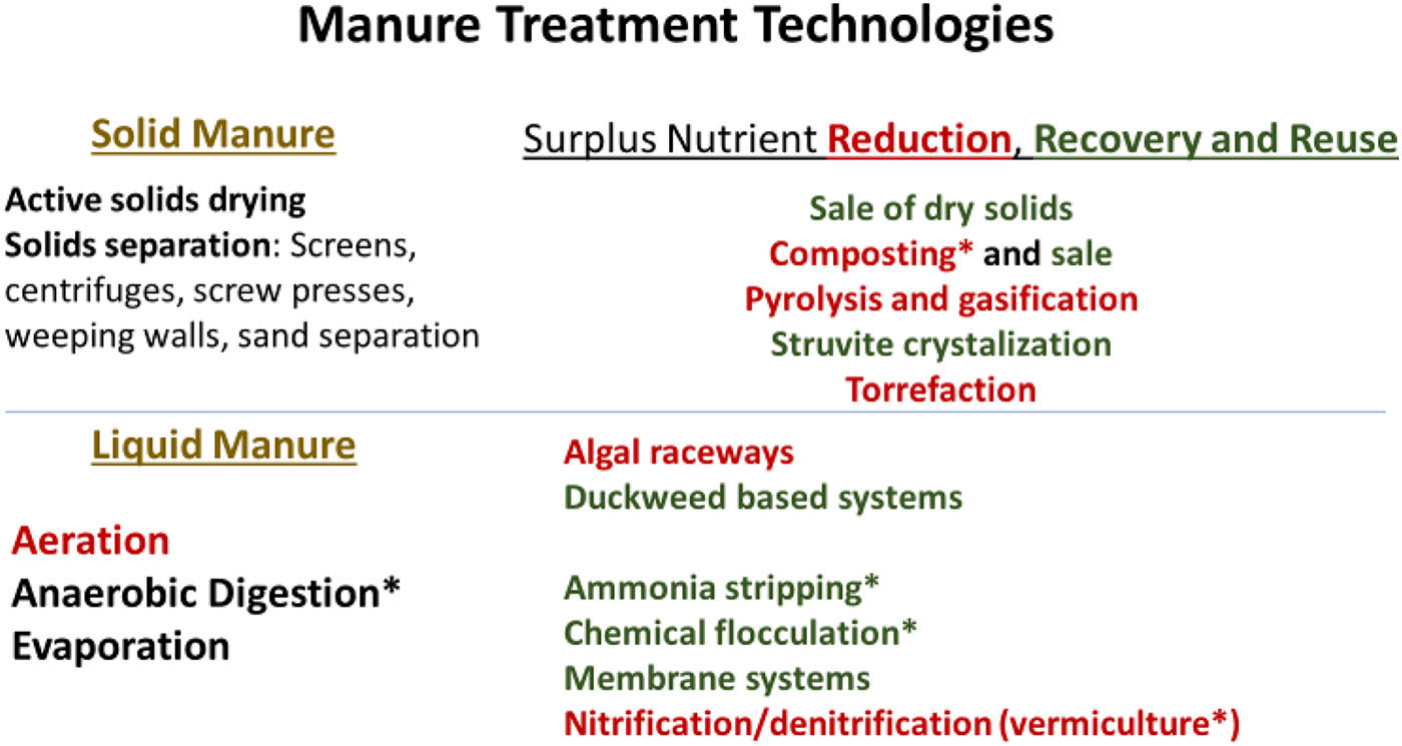
Manure treatment technologies categorized by type. Those discussed are indicated with an asterisk symbol.

Due to economic pressures, changes in technology, increasing cow productivity, and improved understanding of livestock nutritional management, most California dairy farms have increased herd sizes to increase productivity (Njuki, 2022). Intensification sustains farms economically and can improve environmental performance but increases the amount of manure nutrients to be managed on‐site. As herd size increases relative to land available for manure application, N and other nutrients accumulate in amounts eventually exceeding the capacity for on‐farm crop recovery. A mature, large‐breed dairy cow produces approximately 0.45 ± 0.096 kg N cow^−1^ day^−1^ (ASAE, 2005), with a reported range for lactating cows in California of 0.417–0.565 kg N cow^−1^ day^−1^ depending on cow size, breed, stage of lactation, and diet (ASAE, 2005; Chang et al., 2006). Farms with stocking rates (SRs) (milking cows plus replacements) that generate manure nutrients after storage losses in amounts greater than can be recovered and removed by crops accumulate surpluses. Manure application to land at levels beyond crop recovery increases the potential for nitrate leaching to groundwater and atmospheric nitrous oxide (N_2_O) emissions.

Manure is biologically dynamic and undergoes constant transformations based on its dry matter (DM) content, temperature, handling, and storage. These factors influence the amount and type of N conserved in manure applied to crops or available for transport and off‐farm use. Physicochemical characteristics also affect and can limit potential distribution across the landscape. Whether manure application to crops is a net sink for N is a function of crop type, nutrient requirements, N uptake rate and removal, the length of the growing season, and the number of crops per year (Leytem et al., 2021). Exporting surplus N in manures to nondairy farms as a substitute for commercial fertilizers converts this environmentally burdensome by‐product into a potential environmental and economic asset. All dairies in California must operate by permit under the state's General Dairy Order (CVRWQCB, 2025), restricting application of more manure to fields than can be recovered by crops. California waste discharge regulations restrict total N application to an application ratio (AR) of 1.4, or 140% of the N harvested annually in crops. This is assumed to remove at least 71% of applied manure N in crop biomass (Chang et al., 2006; CVRWQCB, 2013). N application beyond the regulatory limit has been associated with groundwater nitrate (NO_3_) loads that exceed the drinking water limit of 10 ppm NO_3_‐N per liter (Dzurella et al., 2012; Harter et al., 2012; Miller et al., 2017; Parsons & Harter et al., 2018). Manure N produced in excess of 1.4 times crop uptake and removal is potentially available for export off farm.

Regulatory guidelines focus on total N in annually applied manure and do not account for specific factors that influence transformation of manure N infield, such as application method, variable manure and soil properties, crop type, and climate. Manure type, application method, and organic N mineralization characteristics are not considered. An AR of 1.4 is considered conservative with respect to the potential for nutrient loss to groundwater to provide a margin of safety (Chang et al., 2006) and remains the most pragmatic approach to manure nutrient management due to large uncertainties associated with application rates, organic matter mineralization, and other factors (Burton, 2007). Nevertheless, discrepancies between reported and calculated N contents in manure at the farm level have been identified (Cativiela et al., 2019; Chang et al., 2006; Cohen‐Davidyan et al., 2020; Miller et al., 2017; Parsons & Harter et al., 2018), amounting to approximately 40% of farm‐level N budgets. These accounting deficiencies explain in part observed nitrate exceedances in groundwater in areas of the Central Valley (CV) with large numbers of dairy farms (Cativiela et al., 2019). Proximity to neighboring dairies or regional concentrations of cows can concentrate N application locally and limit export options yet are often not considered in AR calculations.

Most dairies in California manage some portion of manure using water to flush concrete surfaces in barns and feeding areas, while some manure falls in corrals and pens and is managed in solid form (Meyer et al., 2019; Miller et al., 2017). This portion of the manure accumulated may be treated using novel manure nutrient recovery technologies such as dissolved air flotation (DAF), vermifiltration (VERM), and ammonia stripping and recovery through mechanical vapor recompression (MVR) (Figure 1). These afford opportunities for N extraction and concentration or surplus N reduction while producing low‐moisture products for more economically viable off‐site transport (Kaffka et al., 2022). Previous studies have shown that DAF can effectively recover over 30% of N and 80% of P post liquid‐solid separation (Porterfield et al., 2020). MVR technology is largely still in development stages, but studies investigating soil applications have found N:P ratios of 1.8–4.6, indicating variable recovery efficiencies (Baxter et al., 2024). VERM systems emphasize nutrient reduction via denitrification and recover less N but generate wood chip residues and vermicompost that can be applied as an agricultural amendment (Miito et al., 2021).

A better understanding of the scale and distribution of surplus manure nutrients, especially N, and the potential for technological solutions will support more effective and efficient regulation. The objectives of this paper are (1) to provide spatially resolved, quantitative estimates of surplus N within the dairy sector in high‐priority nitrogen management zones (NMZs) in California's San Joaquin Valley (Figure 2), required to address this problem in the short term, (2) to evaluate selected manure treatment technologies capable of recovering manure N in useful form in terms of N surplus reduction and climate performance, and (3) to support incentive‐based dairy industry solutions for surplus N management and prudent policy development.

**FIGURE 2:**
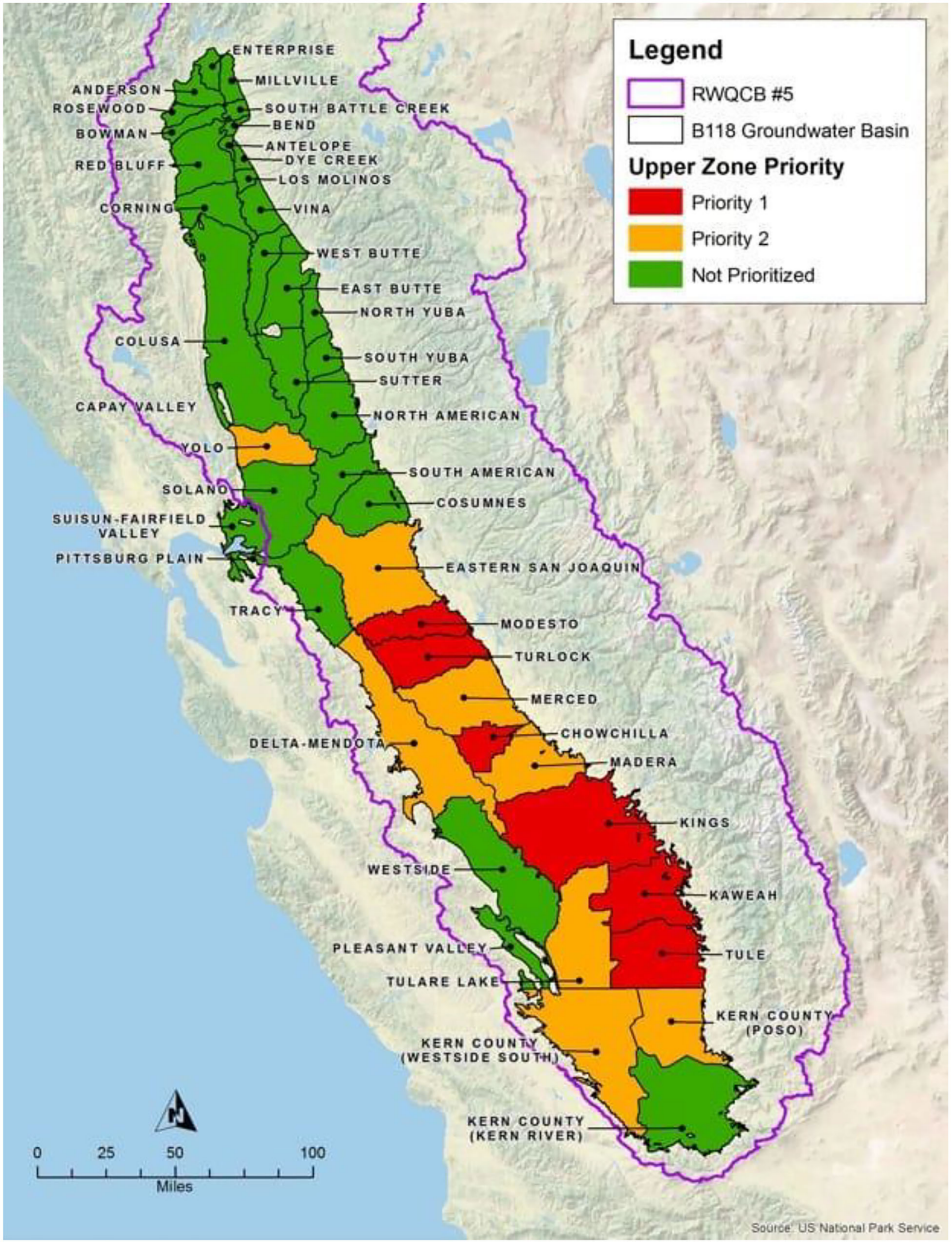
Nitrogen management zones and groundwater sub‐basins (CV‐Salts, 2025).

## 2 MATERIALS AND METHODS

### 2.1 Manure nutrient estimation

Due to the dynamic character of manure N and the diversity of management practices existing on farms, errors are unavoidable when accounting for manure nutrient content. The source and amount of error affects estimates of nutrient surpluses and must be considered when evaluating policies that regulate dairy farms. Oenema et al. (2003) summarized many years of research and experience in the Netherlands with farm nutrient management and accounting. They concluded that nutrient budgets estimated at the farm gate (inputs and outputs) are considered more accurate than budgets estimated from field application and crop uptake measurements. Records of sales of milk and livestock, manure or compost transported off the farm, and purchase of feeds and fertilizers typically are accurately accounted (5% error or less). Measurements within the farm boundary and for fields are less certain. Crop responses prior to planting or early in the season, including uptake and removal of nutrients, vary and can only be estimated. This affects the balance between nutrients applied and crop removal. Even these estimates can be more accurate than budgets based on calculations of soil‐related state variables, transformation processes, and resulting emissions that can vary by 10%–20% or greater (Burton, 2007; Cativiela et al., 2019; Miller et al., 2017; Oenema et al., 2003).

At the NMZ level, average values from the literature most relevant to California conditions were used for estimating manure N surpluses (Kaffka et al., 2022). The fate and character of manure N depends on deposition, collection, and storage. Here both free‐stall and open‐lot structures were considered because they affect the character and nutrient content of collected manure differently. Estimates for the proportion of dairies using free‐stall barns, open lot housing or combinations of the two types were reported by Cohen‐Davidayan et al. (2020) and Meyer et al. (2019) (Figure S1). Free‐stall dairies mainly collect liquid manure (LM) from concrete surfaces to storage lagoons, while open‐lot dairies primarily conserve solid manure (SM) from paved feeding areas and corrals, but both types of dairies have some amount of liquid and SM in differing proportions. Since N conservation and storage losses vary by dairy type—free stall (FS) or open lot (OL)—and since the number of each type in each NMZ was not known, estimates for both FS and OL dairies are considered.

Data including farm location, cattle population, and acreage were acquired and organized by the Central Valley Representative Monitoring Program (CVDRMP, 2025) from nutrient management plans submitted to the Central Valley Regional Water Quality Control Board from 2017 to 2019 (Figures 4 and 5) and used to estimate manure amounts for each dairy (537 farms out of 984 in the available data were located in Priority 1 NMZs). These data were judged to be sufficient for assessment of charges to individual farms for groundwater remediation costs. Some dairies reported raising heifer replacements on separate farms while others raised replacements on the main farm, and/or purchased heifers. To simplify, we assumed that all dairies raised a representative amount of young stock based on Miller et al. (2017) and Harter et al. (2017). Manure from replacements and dry cows was included in the SM fraction.

SRs are the number of mature milking and dry cows plus replacements (calves and heifers) per unit of total land available on‐site for manure application and feed crop production. Farms with larger SRs tend to have larger nutrient surpluses. Our analysis uses SR as the basis for geospatial assessment of surplus nutrient availability and distribution. Equations and parameter values used in calculating surplus manure N and their sources are listed in Tables 1 and 2. When the literature reported differing values, we used values considered reasonable in consultation with CVDRMP members and other industry stakeholders.

**TABLE 1:** Variables, units, values and sources for calculating manure N surpluses.

Variable	Units	Symbol	Value	Source(s)
Manure N per cow + replacements	kg N ha^−1^	MN	200	(Chang et al., 2006; Pettygrove et al., 2009; others)
Stocking rate	# of mature cows per farm per ha receiving manure	SR	1–80	Calculated from CVRWQCB data
Free stall farm (FS): Fraction of MN falling on concrete = liquid manure (MN)	Unitless	FS_LM_	0.6	(Meyer et al., 2019; Cohen‐Davidyan et al., 2020; Harter et al., 2017; Lorimor et al., 2004; other sources)
Fraction of liquid manure conserved	Unitless	LM_con_	0.7	(Meyer et al., 2019; Davidyan, 2021; Harter et al., 2017; Lorimor et al., 2004; other sources)
Free stall farm: fraction of manure N falling in corrals as solid manure (SM)	Unitless	FS_SM_	0.4	By difference; Meyer et al., 2019; Cohen‐Davidyan, 2020; Harter et al., 2017; Lorimor et al., 2004; other sources)
Open lot farm (OL): fraction of liquid manure	Unitless	OL_LM_	0.35	(Meyer et al., 2019; Davidyan, 2021; Harter et al., 2017; Lorimor et al., 2004; other sources)
Open lot: fraction of manure falling in corrals as solid manure	Unitless	OL_SM_	0.65	(Meyer et al., 2019; Davidyan, 2021; Harter et al., 2017; Lorimor et al., 2004; other sources)
MN sold off‐farm	Unitless	OL_SM_	0.2	(Parsons & Harter, 2018)
Hectares receiving MN per farm	ha	ha	From data	CVRWQCB data (2018–19)
N removed by crops (cereal and corn silages)	kg N ha^−1^ yearr^−1^	CR_crop_	450	(Chang et al., 2006; Miller et al., 2017; Pettygrove et al., 2009)
N removed by crops (cereal, corn silages, and Sudan grass hay)	kg N ha^−1^ year^−1^	CR_crop_	630	(Chang et al. 2006; Miller et al., 2017; Pettygrove et al., 2009)
Application ratio of total manure N relative to crop uptake	Unitless	AR	1.4	(CVRWQCB; Chang et al. 2006; Miller et al., 2017; Pettygrove et al., 2009)
Fertilizer N applied to corn silage	kg ha^−1^	FertN	56	Other sources

*Note*: Other sources refer to the personal communications with farmers and consultants in the dairy industry.

**TABLE 2:** Calculations for surplus manure N per dairy farm.

Variable	Equation	Purpose
MN	200 × SR	Total raw manure N per ha for one cow + replacements (kg) based on the stocking rate on each farm in the database
FS_MN_cons_	MN × (FS_LM_ × LM_cons_) + MN × (FS_SM_ × SM_cons_)	Calculates MN available after volatilization losses on a free stall dairy, per cow equivalent
OL_MN_cons_	MN × (OL_LM_ × LM_cons_) + MN × (OL_SM_ × SM_cons_)	Calculates MN available after volatilization losses on an open lot dairy, per cow equivalent
FS_MN_sold_	FS_MN_cons_ × 0.2	Assumes 20% of total manure N (after volatilization losses) is sold
OL_MN_sold_	OL_MN_cons_ × 0.2	Assumes 20% of total manure N (after volatilization losses) is sold
FS_MN_net_	FS_LM_ × LM_cons_ + (FS_SM_ × SM_cons_ − FS_MN_sold_)	Assumes only SM is sold. Deducts total MN_sold_ only from the SM fraction
OL_MN_net_	OL_LM_ × LM_cons_ + (FS_SM_ × SM_cons_ − FS_MN_sold_)	Assumes only solid manure is sold. Deducts total MN_sold_ only from the SM fraction
CROP_N	CR_xcrops_ × AR	MN recovered by crops (*x* = either a two‐ or three‐crop system)
FS_MN_surplus_	(FS_MN_net_ + FertN − CROP_N) × ac	MN left after volatilization losses, sales, and crop removal based on the number of cows per ha and the number of ha per farm
OL_MN_surplus_	(OL_MN_net_ + FertN − CROP_N) × ac	MN left after volatilization losses, sales, and crop removal based on the number of cows per ha and the number of ha per farm

Parsons and Harter (2018) estimated that 20% of California dairy manure on average was sold off‐farm. Attributing this value to all farms implies a large amount of uncertainty, as average values do not reflect actual conditions on individual dairy farms. It is likely that off‐farm sales are not uniformly distributed. High SR farms in general have larger surpluses and may be responsible for a larger portion of off‐farm manure sales and distribution, especially if they have large amounts of SM to manage. Co‐location of nearby dairy farms is also likely to influence the potential for removal of manure nutrients. Reducing manure N through off‐farm distribution increases the SR that is in theoretical balance with crop uptake. As available data do not indicate which farms sell manure nor how much they distribute off‐farm, we have applied the Parsons and Harter (2018) estimate of 20% off‐farm distribution to all farms, even though off‐farm distribution is unlikely to be even across all farms. We have also assumed that the SM fraction is distributed off‐farm, rather than the LM fraction (Tables 1 and 2).

Crop uptake and nutrient removal are key considerations in calculating manure nutrient ARs and surpluses. Chang et al. (2006), UC Committee of Experts, summarized earlier research on manure N management in California. To develop ARs, they evaluated the relationship between crop growth and nutrient uptake for typical dairy crop production systems based on a survey of dairy farm cropping practices and representative yields. Rotations were based on common dairy cropping practices in the CV, which has a year‐round growing season: winter cereal silage crops (wheat/triticale) followed by corn silage in summer. A third late summer Sudan grass or sorghum silage crop was also estimated. Annual crop yields were determined to remove 450–630 kg N ha^−1^ year^−1^, with input requirements in the range of 630–780 kg N ha^−1^ year^−1^ (Chang et al., 2006). Miller et al. (2017), using crop production data from 2012, reported similar values for corn silage nutrient uptake, but larger amounts for cereal silages (200–220 kg N ha^−1^) than used in Chang et al. (2006). They proposed an AR range of 1.4–1.65. The conservative value of 1.4 was adopted by state policymakers and is used in this study.

Fertilizer N is sometimes applied to crops on dairy farms, particularly corn silage, to correct perceived deficiencies in N availability resulting from inadequate mineralization of organic N in manures. Not all farms use fertilizer, and the amounts purchased are uncertain from farm to farm and year to year. Purchased N decreases the SR that can be balanced with crop uptake on a total N basis. We have included a small amount of starter fertilizer N applied to corn silage crops for all farms (56 kg N ha^−1^) because reports in the literature (e.g., Chang et al., 2006; Pettygrove et al., 2009) and discussions with farmers and dairy consultants indicate this is common practice.

### 2.2 Assessment of manure nutrient recovery, reuse, or reduction technologies

Many approaches have been proposed, and some demonstrated to manage surplus nutrients in manures through nutrient recovery or denitrification (Dong et al., 2022; Vanotti et al., 2020). Manure separation technologies are generally categorized by solids separation method, including mechanical separation, filtration, sedimentation, and gravity filtration (Burton, 2007). Each technology is limited in different ways, affecting feasibility for on‐farm use. To develop novel fertilizer products from manure and for accurate accounting it is important to understand how separation systems influence nutrient partitioning into SM or LM fractions. LM is often higher in N content, whereas SM can be higher in P (Meyer et al., 2011; Powell & Wu, 1999), potentially reducing SM value as a mineral N fertilizer replacement.

These technologies are listed in Figure 1, organized as primarily SM or LM treatment systems. Technologies and associated products evaluated were chosen to represent different strategies and because they are either in commercial use, demonstrated on dairies, and/or available in California. These include a DAF/Flocculation technology related to wastewater treatment methods, an evaporation/thermal compression technology partially derived from food processing and adapted for manure (MVR), and a VERM process that primarily reduces N in manure on farm through denitrification, rather than recovering most reactive N. These processes are described in Figures S2–S5 and in greater detail in Kaffka et al. (2022).

Figure 3 depicts the complex, multiple‐stage separation and handling pathways for raw manure to become solid amendment products. Solid by‐products from the technologies evaluated were assessed based on recovered N content. These products were evaluated for potential mineralizable N (PMN) as described in Supporting Information. Results from those analyses were used to estimate their nutrient replacement value as well as for techno‐economic assessment and life cycle assessment (LCA) modeling (Table 3; Figure S4; Figure 6). Calculations and technical details are in Supporting Information 2 and Kaffka et al. 2022.

**FIGURE 3:**
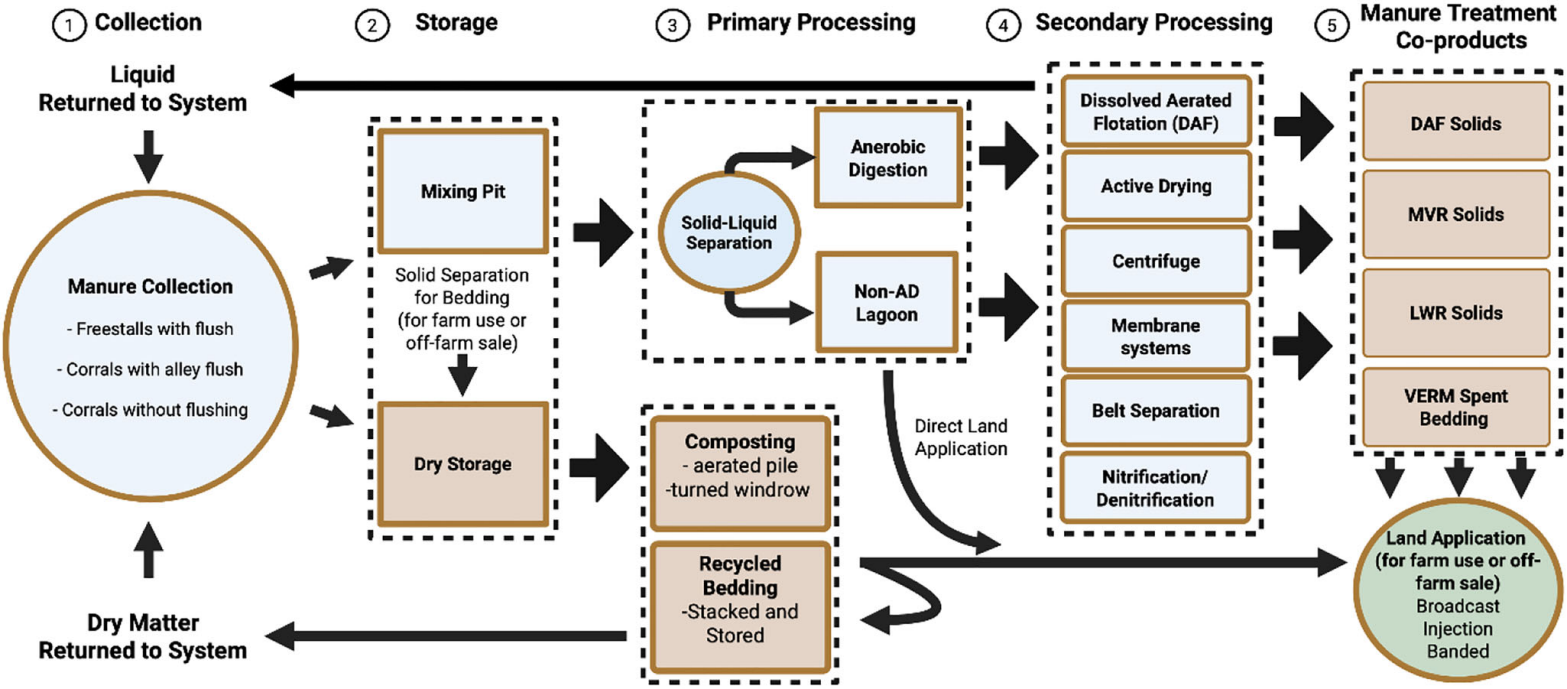
Schematic diagram representing the flow of dairy manure from collection to final processing as novel fertilizer products (created using Biorender).

**FIGURE 4:**
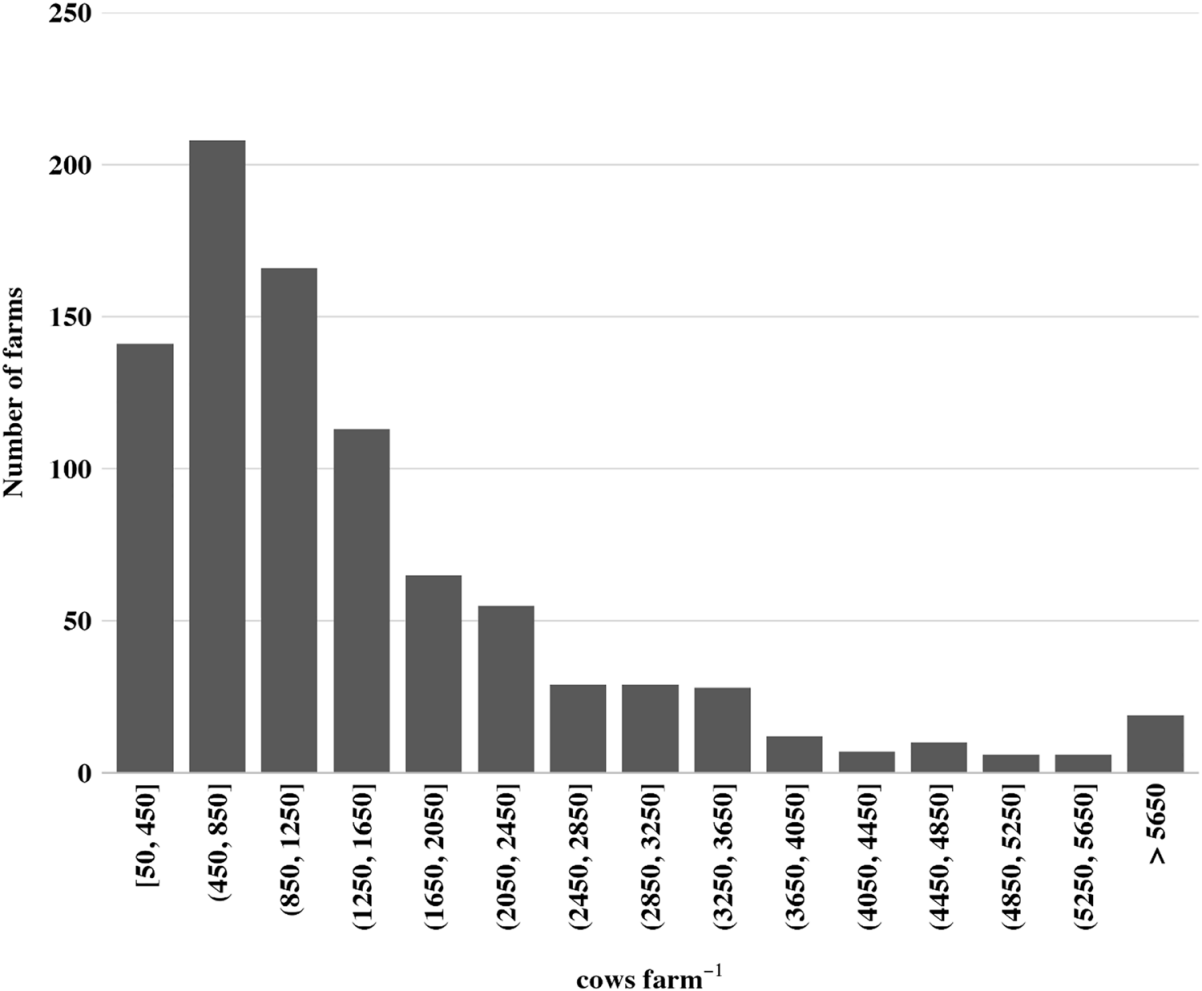
Frequency distribution of dairy farms by herd size, expressed as the number of cows per farm. Bars indicate the number of farms within each herd‐size class (n = 894 farms, including priority one nitrogen management zone [NMZ] farms).

**FIGURE 5:**
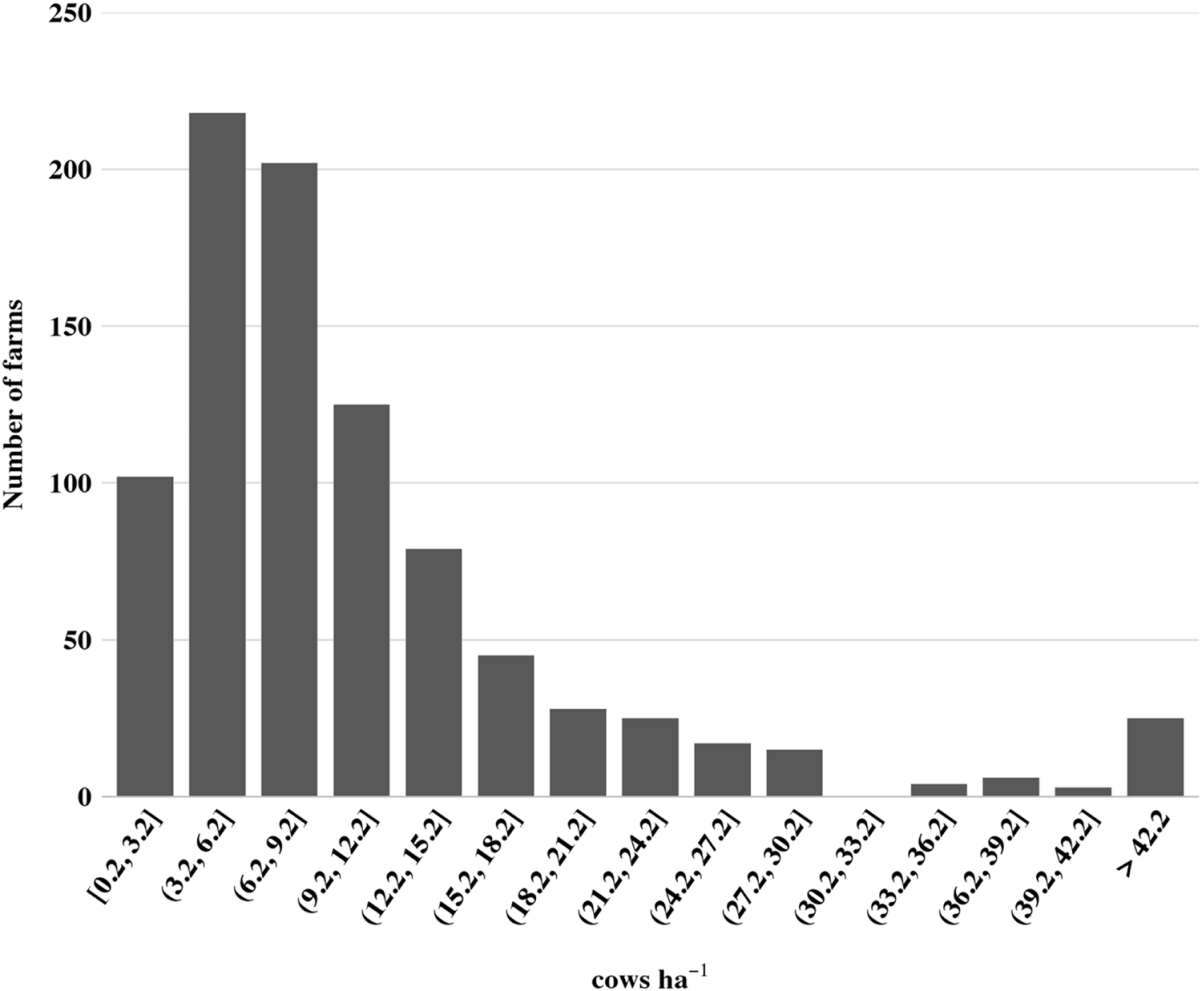
Frequency distribution of dairy farms by stocking density, expressed as the number of cows per hectare. Bars indicate the number of farms within each stocking‐density class (n = 894 farms).

**TABLE 3:** Chemical characteristics of select manure‐derived products.

Parameter	Unit	COM	VER	DAF	LWR	MVR
Total C	%	21.6	96.6	22.6	18.3	34.5
Total N	%	2.6	6.6	3.0	2.0	3.3
NH_4_^+^	mg g^−1^	0.98	1.89	0.84	1.16	1.35
NO_3_^−^	mg g^−1^	0.27	0.39	1.64	0.37	0.25
C/N ratio		8.5	14.5	7.4	9.4	10.4
NH_4_^+^/TN ratio		0.04	0.03	0.03	0.06	0.04
pH		9.3	8.3	6.8	8.5	8.2
EC	mS/m	7.3	2.1	4.5	0.8	17.9

Abbreviations: COM, dairy manure compost; DAF, dissolved aerated flotation solids; LWR, livestock water recycling solids; MVR, mechanical vapor recompression; VER, dairy manure from vermifiltration bedding.

**FIGURE 6:**
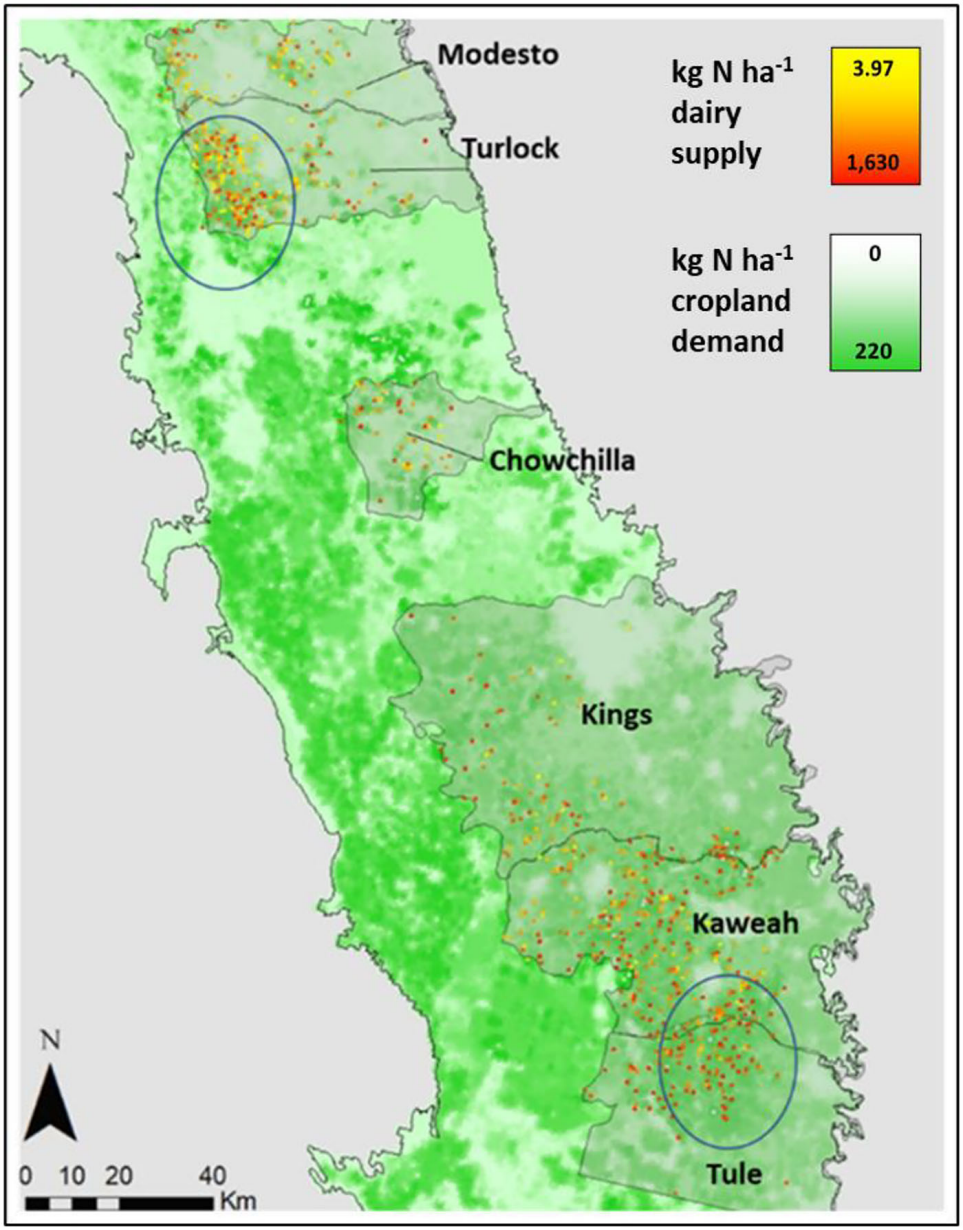
Dairy nutrient concentration and potential demand for surplus nutrients, both on and off‐site. The dairy farm locations in the San Joaquin Valley are mapped by estimated N amounts supplied per 1 km^2^ hexagonal grid (in yellow and red). Areas with dense clusters of farms are likely locations for nutrient recovery and removal systems.

### 2.3 Distribution of manure products

Transfer of dairy nutrient products to regional nondairy cropping systems where they can displace conventional fertilizers adds significant potential demand for freight transport. Geospatial relationships and associated transportation requirements between dairies and available nondairy cropland, accounting for nutrient demand, were estimated using a source‐sink distribution model developed in R (Supporting Information 5, Figure 7). The model utilizes a near distance table relating dairy sources to cropland sinks produced in ArcGIS to calculate transportation requirements. In this analysis, the near distance table of inputs was produced from a 1‐km hexagonal (hex) grid overlay of the CV to which dairy cattle populations, farm locations, manure production, NMZ, and crop N demand were aggregated.

**FIGURE 7:**
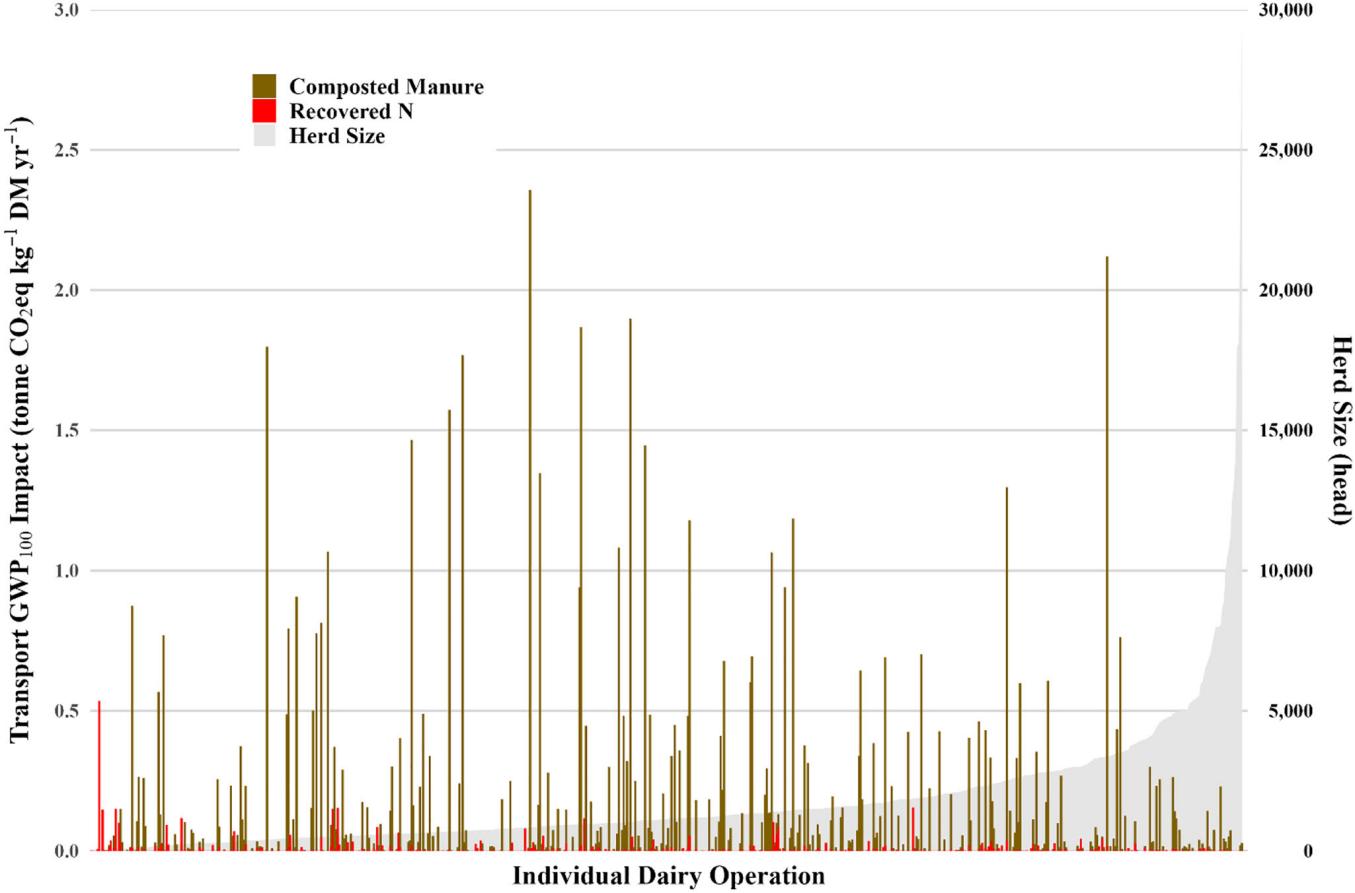
Freight transport impact for distribution of composted manure or generic manure‐derived recovered N fertilizer products from dairy to nondairy cropping systems compared with herd size. Red lines indicate freight transport GWP_100_ impact from delivery of N product to cropland; brown lines indicate GWP_100_ impact for delivery of composted manure to cropland.

Due to an absence of data, PMN was determined for manure compost (COM), VER, livestock water recycling solids (LWR), and MVR solid byproducts using a short‐term 28‐day mineralization assay (Curtin & Campbell, 2006) (Supporting Information 4: Figure 6). The model distributes estimated PMN products from dairy hex to crop hex outside of the Priority 1 NMZs, in order of near rank (closest to farthest) and in amounts proportional to the percentage of total CV nutrient demand represented by the destination 1‐km hex. Materials were distributed based on total available PMN content for each SM product. Crop N demand was based on estimated cropland use from 2018 data (USDA NASS, 2020) and crop fertilizer N rates and application patterns obtained from UC Davis Economic Cost and Return Studies (UCD ARE, 2021). This process was iterated over each source‐sink hex pair by near distance rank and repeated until 99% of estimated nutrient supply was delivered. Crop hexes were removed as possible sink destinations when their nutrient demand was filled.

Annual product transport mass was calculated based on the nutrient mass percentage (N) and moisture content of each specific N product (Table 4), and transport impact reported as metric tonne‐kilometers (tkm) per kg of product. The tonne‐kilometer impact was then converted to tonnes CO_2_eq as GWP_100_ using life cycle inventory (LCI) data from the EcoInvent database and TRACI2.1 impact characterization factors accessed via GaBi‐ts Software (Ecoinvent, 2020; PE International, 2019; USEPA, 2017). These output values are driven by dairy herd size and corresponding manure N output, as well as by the spatial and supply–demand relationships between N sources (dairies) and nondairy cropland N sinks. Manure production, nutrient recovery, cropland demand, and delivery were calculated for a single production year. Dairy sources and cropland sinks were presumed to produce/demand similar quantities of N in any given year, in an ongoing manner.

**TABLE 4:** Nutrient product mass balance by waste treatment system.

Per kg manure dry matter input	DAF system	Mechanical vapor compression system		Vermifiltration system	Anaerobic digestion	Compost/windrow
		Aqueous ammonia distillate	Solid to compost			
Product dry matter (kg)	0.55	0.066^a^	0.98	1.24^b^	–	0.618
N output (kg)	0.0043	0.054	0.029	0.019	0.036	0.049
P output (kg)	0.0013	–	0.013	–	0.042	0.023
K output (kg)	0.0009	–	0.028	–	0.255	–
N replacement value	0.62	1	0.62	0.62	–	0.53

^a^ Solute mass reported for liquid nutrient product.

^b^ Includes addition of woodchips at an assumed 1:1 mass ratio.

*Source*: California Air Resources Board; Hao et al. (2001).

### 2.4 LCA of representative manure treatment technologies

The climate impact of nutrient recovery technologies was estimated using a previously developed ISO14044‐compliant scalable, process‐based, agronomically responsive cropping system LCA (SPARCS‐LCA) model, described in detail in Marvinney and Kendall (2021). Lagoon storage was used as a baseline for consequential assessment of alternative technologies for dairy LM management, with each alternative technology considered additively. The most common alternative is anaerobic digestion (AD). AD systems in California typically use an unheated, covered LM storage lagoon to collect biogas produced under low‐oxygen conditions. Liquid waste influent flows into the AD system following collection from concrete areas in free stall barns, feeding areas, and milk houses after solids are separated. Separated solids are reused for bedding or spread on fields (Kaffka et al., 2022).

Pumping of liquid effluents is excluded from comparative analysis as energy demand was assumed to be identical for each of the technology alternatives and the AD baseline. The biogas produced from AD was assumed in system expansion calculations to displace fossil fuel electricity generation. Manure storage, including residual materials from AD, also results in on‐site emission of methane, nitrous oxide and carbon dioxide as fugitive emissions, calculated as 5% of methane yield (CARB, 2014). Lagoon carbon dioxide emissions, derived from short‐term biogenic sources, are here treated as carbon neutral in keeping with ISO14044 guidelines (Finkbeiner et al., 2006).

In the SPARCS‐LCA model, the three recovery technology scenarios are defined by a unique set of state variables, inputs, and outputs. Each process component represented in a scenario‐specific system diagram is associated with a particular set of input and output flows (i.e., energy and material demand, manure DM, nutrient, and waste output) (Figure S5, Figure 7). These flows are quantified at each stage in the nutrient recovery process and subsequently summed. Impact footprints are reported for each process component and for the whole system. N recovery technologies are compared based on calculated impacts per kg DM input against open lagoon storage and AD baselines.

System expansion is an LCA method by which the benefits of a process output can be calculated by expanding the analytic boundary of the system in question to include processes or products which are displaced or substituted. The system output can then be credited with avoidance of impacts from production of the displaced product. This approach was adopted to calculate the potential benefits of recovered N products derived from displacement of chemical N fertilizer. This displacement benefit was calculated as the N content of nutrient recovery output multiplied by fertilizer replacement values. This quantity was used to calculate chemical flows defined by a US generic N fertilizer production LCI dataset from Ecoinvent, converted to impacts using TRACI2.1 impact factors, and subtracted from the total impacts calculated for each nutrient recovery process. This displacement benefit was calculated as the N content of recovered nutrient output multiplied by fertilizer replacement values. This quantity was used to calculate chemical flows defined by a US generic N fertilizer production LCI dataset from Ecoinvent, converted to impacts using TRACI2.1 impact factors, and subtracted from the total impacts calculated for each nutrient recovery process.

## 3 RESULTS

### 3.1 Surplus N estimates

Table 5 reports average and median numbers of mature cows plus replacements, land available for manure application per farm, and SRs for each NMZ and all dairies in each region. Chowchilla, Modesto and Turlock NMZs are in the Northern San Joaquin Valley (SJV), and Kaweah, Kings, and Tule districts are in the Southern San Joaquin Valley (SSJV). Turlock NMZ has the largest number of dairies and cows. In general, dairies in the Northern San Joaquin Valley are older and tend to have less land area and smaller herd sizes. Farms with larger SRs have the most challenging surplus N management issues. Table S1 reports estimated N surpluses based on SR, using the values and calculations in Tables 1 and 2. These values account for losses during storage and handling based on the references cited and are discussed in greater detail in Kaffka et al. (2022). Manure N on farms with SRs less than or equal to 7–8 cows per ha is approximately in balance with crop requirements in the year‐round growing season of the SJV, assuming volatilization N losses before manure application.

**TABLE 5:** Number of mature cows (lactating and dry) for dairies within Priority 1 and 2 nitrogen management zones (NMZs) (cows per ha).

Location	# of farms	Mature cows		Ha		Stocking rate	
		AVE	Median	AVE	Median	AVE	Median
All dairies	84	1458	1070	224	126	11.5	8.2
Chowchilla	32	1685	1103	369	224	7.4	4.6
Modesto	53	880	700	107	82	9.9	8.2
Turlock	182	980	1057	117	119	12.4	9.4
Kaweah	116	1580	1200	229	187	12.4	8.2
Kings	119	1730	1379	304	205	12.4	7.7
Tule	101	2280	1789	314	222	12.4	8.8

*Note*: Based on CVRWQCB data for 2018–2019. Priority 2 dairies and those outside defined NMZs are included in the all dairies category.

Averaging the surpluses in this class of farms and averaging two and three crop systems (equal to high‐yielding crops in a two‐crop system), results in a surplus of 150 kg N ha^−1^ on free stall (FS) farms and 70 kg N ha^−1^ on open lot (OL) farms—a range addressable with currently available strategies such as modestly increased manure export, less LM storage, or cropping system intensification (Kaffka et al., 2022). SRs greater than 9–10 cows ha^−1^ produce surplus nutrients amount greater than potential crop recovery in high‐yielding CV two‐crop per year systems at the allowable AR of 1.4. Table S2 compares SR with surpluses estimated in Table S1 for each NMZ.

### 3.2 Differences among NMZs

SR variability, spatial distribution, and manure management system affect the potential and choice of alternative nutrient recovery and reuse systems. We observed large differences among NMZs with respect to farm size distribution and surplus N. Table S3 provides greater detail on SR distribution within each NMZ. The Turlock NMZ has more total dairy farms and the largest number of high SR and smaller herd size farms. Discussions with industry stakeholders suggest that most of these farms are FS. OL farms are reportedly more common in the SSJV. The Turlock NMZ and Tule NMZ provide useful contrasts. Turlock NMZ has the largest calculated N surplus. This increases marginally, assuming the majority of farms use FS structures with larger amounts of N conserved in LM (Tables S4a and S5a). In contrast, the Tule NMZ has nearly the same number of cows but distributed on fewer and mostly larger farms, and a larger cropland acreage receiving manure (Tables S4b and S5b). Dairies developed earlier in Turlock than in the Tule NMZ in general and reflect different development pathways. Some smaller dairies expanded herd size without adding cropland. Stocking rates are very high on such farms, and small operations (<120 ha) account for a majority of surplus N. Twenty‐three dairies reported SRs of 25 or greater, accounting for an estimated 38% of surplus manure N (Table S4a). All of these dairies reported less than 120 ha of land receiving manure, and more than half reported less than 80 ha (Table S4a). In contrast, the Tule NMZ has approximately half as many total farms with SR greater than 10, and very few are small in size (Table S4b).

### 3.3 Dairy collocation and proximity to nondairy cropland

Surplus N problems are compounded by dairy collocation (Figure 6). The western Turlock NMZ hosts a large dairy cluster. Similarly, an area adjoining the Kaweah/Tule boundary also hosts a large cluster. The proximity of dairy farms to nondairy farmland affects surplus nutrient management opportunities. The transport distance and the character of manure products affect the economic and environmental efficiency of their use (Figure 7). Kaweah and Tule NMZ SM surpluses result in up to a fourfold greater transport impact due to regional clustering and reduced nearby nondairy cropland (Figure 8). Variability in cropland distribution and N demand translates to significant overall differences in N product distribution greenhouse gas (GHG) impacts among the NMZs examined. For example, a cluster of dairy operations in the Turlock NMZ represents a significant N surplus hotspot. This localized excess, however, does not result in equivalently large N product distribution impacts due to the large amount of nearby cropland N‐demand that provides a potential sink for exported N within a relatively smaller transport distance (Figures 6 and  8). Effectively, the large surplus N amounts in the Turlock NMZ are potentially offset by closer nondairy cropland access. Similarly, other NMZs with less surplus N may have larger GHG emissions for SM nitrogen product distribution due to larger distances to suitable nondairy cropland. These differences underscore that solutions to the surplus N problem must be locally adapted and likely will vary between and within NMZs.

**FIGURE 8:**
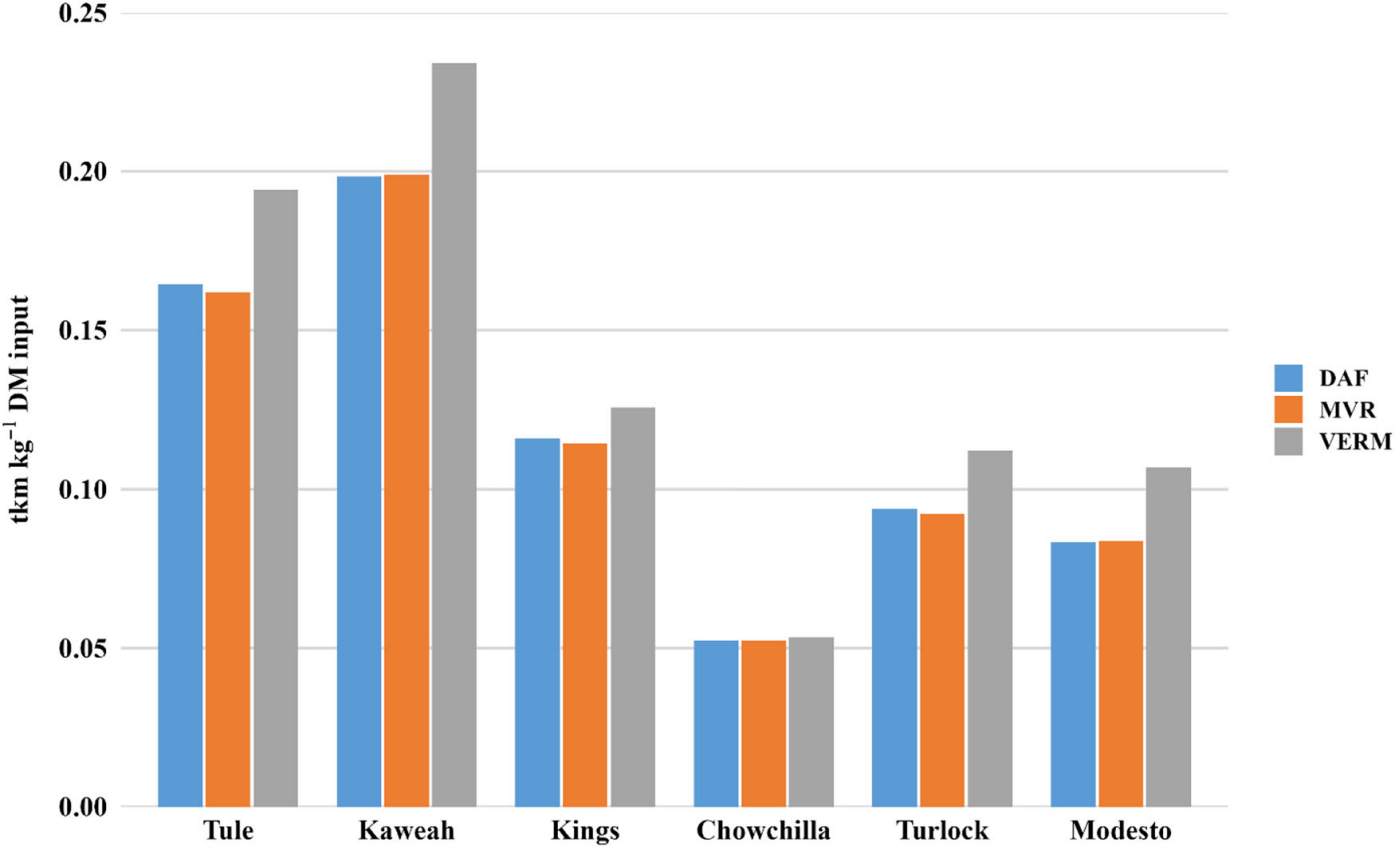
Freight transport requirement for recovered N products by nitrogen management zone, and nutrient recovery technologies. Bars show tonne‐kilometers of transport per kilogram of DM input for Tule, Kaweah, Kings, Chowchilla, Turlock and Modesto.

Estimates of optimum pathways for surplus manure N distribution based on GHG emissions further illustrates the regional character of the surplus N management problem in these NMZs (Figure 9). Regional differences among NMZs are driven primarily by total dairy N surpluses, while variability in N distribution plays a minor role in the total GHG footprint. Addition of DAF and VERM processes to lagoon or AD systems produces additional N products while reducing potential N loading to fields and aquifers, but has minimal effect on system GHG emissions.

**FIGURE 9:**
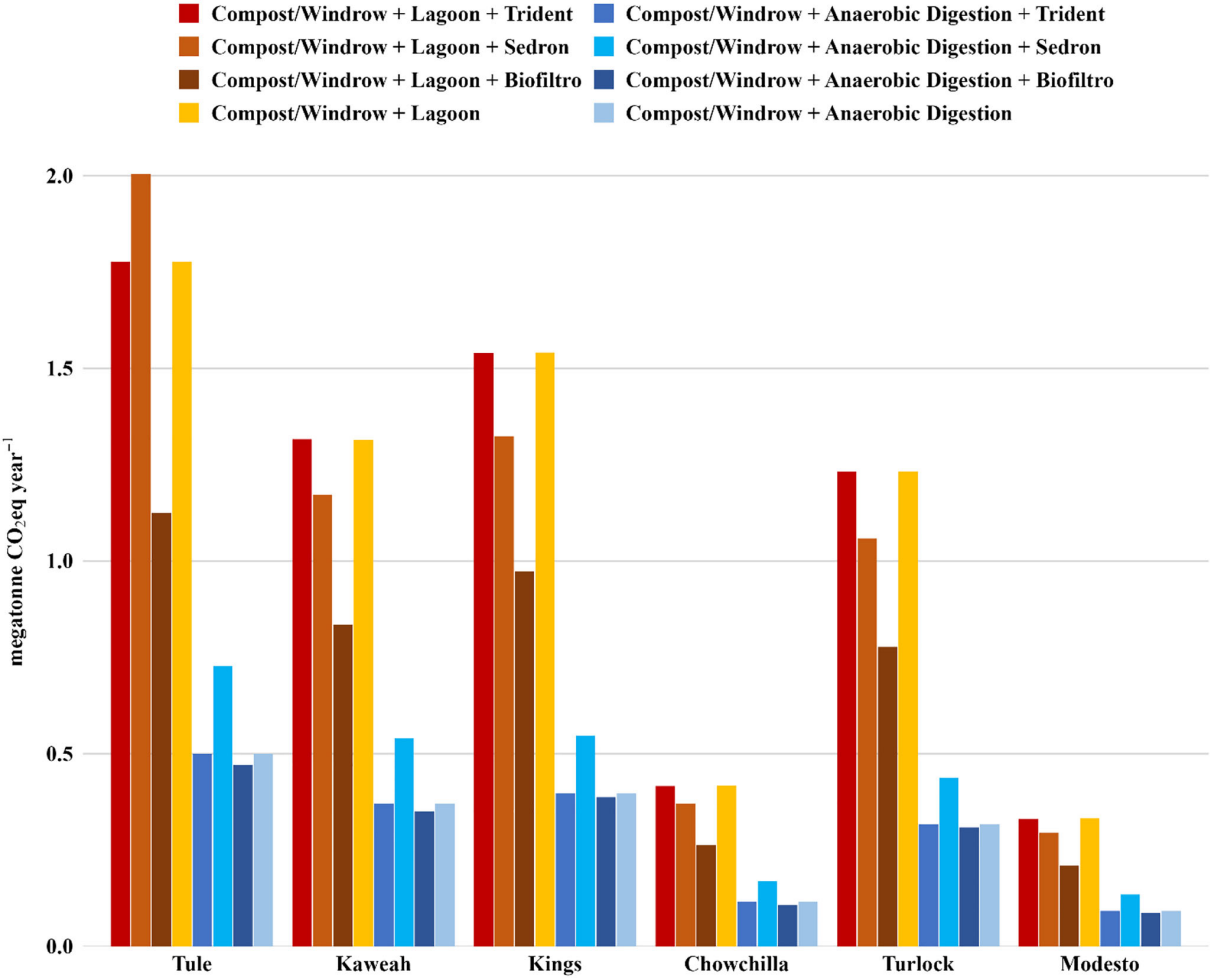
Total estimated GHG emission as Mg CO_2_eq emission under different dairy waste management‐nutrient recovery scenarios by nitrogen management zone (NMZ). The first four columns in each NMZ category represent uncovered lagoon‐based processes, while the second four columns include anaerobic digestion systems.

GHG values associated with manure transport are calculated as the average for byproducts from DAF, MVR, and VERM (Figure 7). Consistent with other studies evaluating transporting of manure in raw or semi‐processed forms, there are reduced transportation emissions associated with recovered N products (red) compared with the same amount of N delivered as composted manure (brown). There was no apparent relationship between dairy herd size (gray) and transportation impacts. Local distribution of nondairy cropland relative to dairy location was the primary factor influencing GHG emissions from recovered dairy N distribution, and a large source of uncertainty in the LCA model. Concentrating SM materials in higher value byproducts with better fertilizer N replacement values supports broader distribution both economically and environmentally (Figure 7).

### 3.4 GHG effects of manure treatment systems

Modeled changes in the GHG emissions of dairy manure management with adoption of each nutrient recovery technology were calculated and output as kg carbon dioxide equivalent per kg manure DM input (kg CO_2_eq kg^−1^ DM). We found that on a 100‐year time horizon for global warming potential (GWP_100_), the DAF system had the lowest GHG footprint at −0.003 kg CO_2_eq kg^−1^ DM. The VERM system produced 0.02 kg CO_2_eq kg^−1^ DM as GWP100, but this impact increased to 0.1 kg CO_2_eq kg^−1^ DM as GWP20, due to estimated release of methane from VERM substrate. The GHG impact of the MVR system depended on influent percent dissolved solids (DS), resulting in 0.91–1.48 kg CO_2_eq kg^−1^ DM at 3.7%– 0.6% DS, respectively.

However, these systems are not designed to be used in isolation, but in combination with lagoon storage and/or anaerobic digestion. Normalized to a lagoon baseline (assumed to produce 8.63 kg CO_2_eq kg^−1^ DM), DAF reduced emissions by 0.09%, and VERM increased emissions by 0.59%. The MVR system increased emissions by 29%–47% over lagoon baseline, again depending on DS content of influent. When used to supplement anaerobic digestion (AD), the combined AD + MVR reduced emissions by 48%–66%, AD + DAF system reduced emissions by 95.6%, and AD + VERM outperformed both, with emissions reduced from lagoon baseline by up to 96.9%.

## 4 DISCUSSION

There are many sources of uncertainty when estimating manure nutrient surpluses on farms. These include the physical/chemical character of the manure, variations in management within and among farms, seasonal variation, and practical limits on quantification of manure nutrients collected and applied. Nutrient accounting is most accurate at the farm gate, where inputs and outputs can be measured and may be as low as 5% (Oenema et al., 2003). Quantifying N movement within farms is subject to larger errors (10%–20% or greater) (Burton, 2007; Cativiela et al., 2019; Chang et al., 2006; Parsons & Harter, 2018). These limitations must be considered when addressing pathways and policies for improved management of manure nutrients. We regarded estimates based on regional average values for relevant parameters as sufficiently accurate to characterize the scale and location of manure nutrient surpluses in the areas of the San Joaquin Valley studied. The level of uncertainty associated with these estimates, however, makes them insufficient to guide specific improvements at the level of an individual farm or to guide on‐farm investments in expensive, technical nutrient recovery systems. These require detailed accounting and testing at the farm level (Cativiela et al., 2019).

Farms requiring potentially expensive investments in advanced nutrient recovery technologies are generally those which exhibit high SRs, limited land for manure application, co‐location near other dairies with surpluses, and/or at large distances from nondairy farmland suitable for bulky SM transport and application. The best technology must be based on the unique characteristics of each farm, including dairy location and potential markets for SMs and nutrient recovery products.

GHG mitigation is an additional consideration and adds constraints on nutrient recovery technologies. While the primary purpose of nutrient recovery technologies is recovery and utilization of excess nutrients (here N) in dairy manures, GHG impacts should be considered nonetheless given the state's aggressive GHG reduction goals.

The alternative manure treatment technologies evaluated vary with respect to net GHG emissions reductions or increases compared to current manure management practices. The VERM system reduces effluent N by denitrification, substantially lowering lagoon GHG emissions if directly treating flushed manure or AD system effluent. However, as a nitrification–denitrification process, VERM loses a large amount of reactive manure N as N_2_ gas, reducing N output for fertilizer replacement (S1.2). VERM requires limited inputs other than energy costs for woodchip production and distribution and post‐use composting but produces methane and nitrous oxide emissions from these processes that negate the GHG benefits of displacing fertilizer with recovered N products. VERM also reduces effluent volatile solid (VS) content and subsequent methane emissions. Assuming an 80% VS reduction, total methane emission as CO_2_eq is reduced by up to 29% (Table S6).

DAF technology concentrates N and other nutrients, improving potential agronomic use (Porterfield et al., 2020), supporting export over greater distances while reducing GHG emissions (Feng et al., 2023). DAF N recovery can protect groundwater from nutrient overloading while also slightly (<1%) reducing net life cycle GHG emissions (Table S6). The DAF system removes most of the fine solids and recovers moderate amounts of N and almost all P in the manure flow. There are potential economies of scale, supporting use on larger dairies or for clusters of smaller dairy farms. The DAF system also supports regulatory limitations on N application and creates a small reduction in GHG emissions compared with the baseline scenario of lagoon storage of LM wastes (Table S6). Stacey et al. (2021) found that costs per kg N can be reduced ∼80%–90% by adopting DAF technology.

The MVR system is effective at recovering nearly all N, P, and K in treated manure, but results in larger GHG emissions than VERM and DAF (Table S6) (Baxter et al., 2024). This process requires significantly larger energy inputs such as electricity and natural gas, while producing a relatively low nutrient density aqueous ammonia product. This system also produces a solid byproduct which is assumed to be composted, resulting in additional GHG emissions from composting. Direct emissions from composting plus the system's required fossil energy use result in a GHG footprint for MVR approximately 11 and 16 times greater than for DAF and VERM, respectively. However, the GHG emissions for N recovery on a per kg DM input basis depend greatly on the solids content of the system's influent, exhibiting increases of 32% and 63% for GWP_20_ and GWP_100_, respectively, when influent solids are reduced from the assumed value of 3.7%–0.6% by mass (Table S6).

Analysis of the DAF system indicates that some GHG emissions can be avoided through displacement of fossil fuel‐based N fertilizer production in the N cake product when calculated using system expansion (Table S6). This displacement benefit slightly outweighs the emissions from system operation and distribution of the N‐enriched cake byproduct. This results in a net negative GHG footprint for the DAF system and leads to a reduction of GHG emissions when this N recovery technology is added to both the baseline lagoon and where anaerobic digestion systems are installed. The MVR system uses significantly more energy and releases methane associated with composting needed for the solid product (Figure 9). Although these emissions are partially offset by fertilizer displacement from use of aqueous ammonia distillate, as well as organic N in the composted solids, the net GHG footprint of this system is positive (Figure 9). This results in a small increase in total GHG emissions with the addition of the MVR system to lagoon and AD storage processes (Figure 9).

VERM fulfills the combined goals of reduced aquifer N loading and nutrient recovery without significant increase in GHG emissions (Figure 9, Table S6) when used upstream of storage lagoons, primarily by removal of volatile solids from treated influent and by reduction in subsequent lagoon methane emissions. VERM also requires relatively low energy inputs and utilizes a waste stream of wood chips. This reduction in downstream emissions is the most important contributor to net emissions, significantly greater than the limited contribution of displaced emissions from avoided fertilizer production.

Tradeoffs occur, for example, if deployed ahead of AD systems, reduction of volatile solids reduces methane production and displacement by renewable natural gas (RNG) of fossil natural gas for transportation fuels or electricity production from RNG. VERM results in the largest total N losses from the manure system through denitrification, reducing the opportunity to recycle reactive N, but simultaneously reduces N_2_O and NH_3_ emissions.

Accurate estimates for the full economic costs of many new alternative manure N management and/or recovery technologies are unavailable or influenced by diverse local factors, differences in policies, subsidies, and stages of development and deployment. New systems typically have higher costs for first of a kind demonstration projects than are ultimately required once working models have become more widely adopted. Projects installed in the United States have all been subsidized for the most part, and markets have not yet discovered the prices that can be accommodated on commercial dairy farms. Policy choices that affect N and other nutrient surpluses in California and elsewhere in the United States are in flux and will have a significant effect on the cost to dairy farmers of new systems. For example, newly installed VERM systems in California are subsidized in part through the state's Alternative Manure Management Program (AMMP) and through sale of carbon credits from methane emission reduction into the state's Cap and Trade System, which is unique to California. Under these circumstances, discussion of manure N recovery system cost is premature and otherwise beyond the scope of this analysis. The Newtrient organization's solutions catalogue provides some current estimates of capital and operating costs for several manure treatment systems or system components, in part based on National Resource Conservation Service cost‐share figures listed by state (NRCS, 2026) and based on their own research (Newtrient, 2026).

The technologies reviewed here and other potential systems (Newtrient, 2025) offer a way to create manure by‐products with valuable use elsewhere in agricultural systems, including for soil health, but come with potentially high capital and operating costs, or loss of otherwise useful reactive N that could be recycled more widely. Achieving adoption of novel N recovery systems on dairy farms requires creative policy incentives that acknowledge high initial costs and financial risk. Creating such policies without forcing farms out of business or resulting in further consolidation within the industry remains a significant challenge.

The Netherlands and Denmark have developed detailed regulations for dairy manure management (Overheid.nl, 2025; Sommer & Knudsen, 2021). Like California, these emphasize restrictions on the amount of manure N that can be applied per ha but also require that surplus manure nutrients be removed and applied off farm. As in this study, these policies are supported by geospatial analyses, which demonstrate the landscape‐level context driving both environmental impacts and the potential productive utilization of dairy nutrient surpluses (Van Apeldoorn et al., 2013). The Netherlands especially has a significant dairy industry with large amounts of surplus nutrients but continues to struggle with defining and implementing policies that further reduce emissions, including NH_3_, P, and nitrates, without resulting in farm bankruptcies or mandated closure (Splinter & Peerlings, 2023; van Grinsven & Bleeker, 2016). Including GHG reduction effects adds a further challenge to successful policy formation.

California has developed successful policies which address methane emissions from manure storage that provide financial incentives for dairy operators and secondary technology providers to develop methane capture and use systems, or for methane reduction, on a competitive grants basis (CDFA 2026a, 2026b). Lee and Sumner (2018) calculated that the capital cost of installing AD systems could equal or exceed the value of livestock on many dairy farms in California, creating unmanageable financial costs and policy risks for most dairies. The Dairy Digester Research and Development Program addresses this risk by subsidizing some of the costs of methane reduction and reuse using AD, incentivizing the use of private matching capital (CDFA, 2026a). Similarly, the AMMP focuses on conversion of LM to SM storage, but now also includes support for the adoption of VERM systems and other related technologies on qualifying dairy farms using a competitive grant approach (CDFA, 2026b, 2026c). These two programs are potential models for similar efforts to address the surplus nutrient problem on California dairies within an otherwise successful and increasingly efficient agricultural sector.

## 5 CONCLUSIONS

This analysis directly supports the need for an integrated approach to policy planning that considers not only estimated manure N surpluses, but also the character of alternative manure products, transport distances, and regional crop N uptake capacities (Yin et al., 2025). Annually, more than half of the dairy farms within regions of the California's San Joaquin Valley classified as high‐priority nitrogen management zones accumulated more manure N after losses due to storage and handling than can be recovered by crops after on‐site application to cropland. Smaller dairy farms tended to accumulate more surplus nutrients than larger farms in general, though some larger farms also reported higher SRs and surpluses.

Manure N surpluses were unevenly distributed geospatially within and between NMZs. Areas with large numbers of collocated farms have correspondingly larger manure N surpluses requiring additional management.

Technical solutions will be affected by dairy colocation, the physical characteristics of individual farms affecting the type of manure management that occurs (free stall, open lot, or mixed types), the character of manure products produced by new management technologies, and distance to nondairy cropland able to receive manure products as fertilizer substitutes. There likely will be multiple optimal solutions, so diverse, creative adaptations should be incentivized by regulatory policy.

Application of life cycle assessment evaluating the GHG emissions of nutrient recovery technologies integrates N recovery and reuse from dairy manure with California's focus on climate mitigation. By considering LCA‐based GHG emission estimates with potential, groundwater protection benefits, tradeoffs associated with new technology adoption can be included in policy‐making processes.

## AUTHOR CONTRIBUTIONS

**Stephen R. Kaffka**: Conceptualization; data curation; formal analysis; funding acquisition; investigation; methodology; project administration; supervision; writing—original draft; writing—review and editing. **Cole Smith**: Data curation; formal analysis; investigation; methodology; writing—original draft; writing—review and editing. **Elias Marvinney**: Formal analysis; investigation; writing—original draft; writing—review and editing.

## CONFLICT OF INTEREST STATEMENT

The authors declare no conflicts of interest.

## Supporting information



Supplementary Material

## References

[jeq270292-bib-0001] ASAE . (2005). Manure production and characteristics (ASAE D384.2 MAR2006). American Society of Agricultural Engineers.

[jeq270292-bib-0002] Baxter, A. E. , Leytem, A. B. , Liptzin, D. , Bierer, A. , & Afshar, R. K. (2024). Effect of dairy manure‐based fertilizers on nitrous oxide emissions in a semi‐arid climate. Soil Science Society of America Journal, 88(6), 2181–2193. 10.1002/saj2.20751

[jeq270292-bib-0055] van Grinsven, H. , & Bleeker, A. (2016). Evaluation of the Manure and Fertilisers Act: 2016. PBL Netherlands Environmental Agency, https://www.pbl.nl/uploads/default/downloads/pbl‐2017‐evaluation‐of‐the‐manure‐and‐fertilisers‐act‐2016‐2779.pdf

[jeq270292-bib-0003] Burton, C. H. (2014). The potential contribution of separation technologies to the management of livestock manure. Livestock Science, 112(3), 208–216. 10.1016/j.livsci.2007.09.004

[jeq270292-bib-0004] CARB . (2014). Compliance Offset Protocol Livestock Projects: capturing and destroying methane from manure management systems . CARB. https://ww2.arb.ca.gov/sites/default/files/barcu/regact/2014/capandtrade14/ctlivestockprotocol.pdf

[jeq270292-bib-0005] Cativiela, J. P. , Angermann, T. , & Dunham, T. (2019). Summary representative monitoring report (revised) . Central Valley Dairy Representative Monitoring Program. https://leadershipcounsel.org/wp‐content/uploads/2019/10/Dairy‐report.pdf

[jeq270292-bib-0006] CDFA . (2026a). Dairy Digester Research and Development Program (DDRDP) . California Department of Food and Agriculture. https://www.cdfa.ca.gov/oars/ddrdp/

[jeq270292-bib-0007] CDFA . (2026b). Alternative Manure Management Program . California Department of Food and Agriculture. https://www.cdfa.ca.gov/oars/ammp/

[jeq270292-bib-0008] CDFA . (2026c). Dairy Plus Program . California Department of Food and Agriculture. https://www.cdfa.ca.gov/oars/dairyplus/

[jeq270292-bib-0009] Chang, A. , Hater, T. , Letey, J. , Meyer, D. , Meyer, R. D. , Campbell‐Matthews, M. , Mitloehner, F. , Pettygrove, S. , Robinson, P. , & Zhang, R. (2006). Managing dairy manure in the central valley of California (UC ANR Publication No.: 9004). UC Committee of Experts on Dairy Manure.

[jeq270292-bib-0010] Cohen‐Davidyan, T. , Meyer, D. , & Robinson, P. (2020). Development of an on‐farm model to predict flow of fecal volatile solids to the liquid and solid handling systems of commercial California dairy farms. Waste Management, 109, 127–135. 10.1016/j.wasman.2020.04.018 32408096

[jeq270292-bib-0011] Curtin, D. , & Campbell, C. A. (2006). Mineralizable nitrogen. In M. R. Carter & E. G. Gregorich (Eds.), Soil sampling and methods of analysis (2nd ed.). CRC Press.

[jeq270292-bib-0049] CVDRMP . (2025). Central Valley Repsentative Monitoring Program. Nitrate Control Program. www.cvdrmp.org/nitrate-control-program.html

[jeq270292-bib-0013] CVRWQCB . (2013). Confined animal facilities‐reissued waste discharge requirements general order for existing milk cow dairies . Central Valley Regional Water Quality Control Board. https://www.waterboards.ca.gov/centralvalley/board_decisions/adopted_orders/general_orders/r5‐2013‐0122.pdf

[jeq270292-bib-0014] CVRWQCB . (2025). Confined animal facilities‐dairy program regulations and requirements . Central Valley Regional Water Quality Control Board. https://www.waterboards.ca.gov/centralvalley/water_issues/confined_animal_facilities/program_regs_requirements/dairy/

[jeq270292-bib-0012] CV Salts . (2025). Central Valley Salts and Nitrates Program. https://www.cvsalinity.org/

[jeq270292-bib-0015] Dong, R. , Qiao, W. , Guo, J. , & Sun, H. (2022). Manure treatment and recycling technologies. Circular economy and sustainability (pp. 161–180). Elsevier.

[jeq270292-bib-0016] Dzurella, K. N. , Darby, J. , De La Mora, N. , Fryjoff‐Hung, A. , Harter, T. , Hollander, A. D. , Howitt, R. , Jensen, V. B. , Jessoe, K. K. , King, A. M. , Lund, J. R. , Medellín‐Azuara, J. , Pettygrove, G. S. , & Rosenstock, T. S. (2012). Addressing nitrate in California's drinking water with a focus on Tulare Lake Basin and Salinas Valley groundwater (Technical Report 3). Center for Watershed Sciences, University of California at Davis. http://groundwaternitrate.ucdavis.edu

[jeq270292-bib-0017] Ecoinvent . (2020). Home page . Ecoinvent. https://ecoinvent.org

[jeq270292-bib-0018] Feng, X. , Smith, W. , & VanderZaag, A. C. (2023). Dairy manure nutrient recovery reduces greenhouse gas emissions and transportation cost in a modeling study. Frontiers in Animal Science, 4, 1134817. 10.3389/fanim.2023.1134817

[jeq270292-bib-0058] Finkbeiner, M. , Inaba, A. , Tan, R. , Christiansen, K. , & Kluppel, H. J. (2006). The new international standards for life cycle assessment ISO 14040 and ISO 14044. The Internatinal Journal of Life Cycle Assessment, 11(2), 80–85.

[jeq270292-bib-0057] Hao, X. , Chang, C. , Lamey, F. J. , & Travis, G. R. (2001). Greenhouse gas emissions during cattle feedlot manure composting. Journal of Environmental Quality, 30(2), 376–386. 10.2134/jeq2001.302376x 11285897

[jeq270292-bib-0019] Harter, T. , Dzurella, K. , Kourakos, G. , Hollander, A. , Bell, A. , Santos, N. , Hart, Q. , King, A. , Quinn, J. , Lampinen, G. , Liptzin, D. , Rosenstock, T. , Zhang, M. , Pettygrove, G. S. , & Tomich, T. (2017). Nitrogen fertilizer loading to groundwater in the Central Valley . California Department of Food and Agriculture and University of California Davis.

[jeq270292-bib-0020] Harter, T. , Lund, J. R. , Darby, J. , Fogg, G. E. , Howitt, R. , Jessoe, K. K. , Pettygrove, G. S. , Quinn, J. F. , Viers, J. H. , Boyle, B. , Canada, H. E. , DeLaMora, N. , Dzurella, K. N. , Fryjoff‐Hung, A. , Hollander, A. D. , Honeycutt, K. L. , Jenkins, M. W. , Jensen, V. B. , King, A. M. , … Rosenstock, T. S. (2012). Addressing nitrate in California's drinking water with a focus on Tulare Lake Basin and Salinas Valley groundwater . Center for Watershed Sciences, University of California. http://groundwaternitrate.ucdavis.edu

[jeq270292-bib-0021] ISO (International Organization for Standardization) . (2006). ISO 14040: Environmental management—Life cycle assessment—Principles and framework. ISO/TC 207/SC 5. International Organization for Standardization.

[jeq270292-bib-0022] Kaffka, S. , Williams, R. B. , Marvinney, E. , & Smith, C. (2022). Manure nutrient recovery, removal and reuse on California dairies . California Department of Food and Agriculture. https://www.cdfa.ca.gov/oefi/research/docs/cbc_manure_nutrient_report.pdf

[jeq270292-bib-0053] van Apeldoorn, D. F. , Kempen, B. , Sonneveld, M. P. W. , & Kok, K. (2013). Co‐evolution of landscape patterns and agricultural intensification: An example of dairy farming in a traditional Dutch landscape. Agriculture, Ecosystems & Environment, 172, 16–23. 10.1016/j.agee.2013.04.002

[jeq270292-bib-0023] Lee, H. , & Sumner, D. (2018). Dependence on policy revenue poses risks for investments in dairy digesters. California Agriculture, 72(4), 226–235.

[jeq270292-bib-0024] Leytem, A. B. , Williams, P. , Zuidema, S. , Martinez, A. , Chong, Y. L. , Vincent, A. , Vincent, A. , Cronan, D. , Kliskey, A. , Wulfhorst, J. D. , Alessa, L. , & Bjorneberg, D. (2021). Cycling phosphorus and nitrogen through cropping systems in an intensive dairy production region. Agronomy, 2021–11, 1005. 10.3390/agronomy11051005

[jeq270292-bib-0050] Lorimor, J. , Powers, W. , & Sutton, A. (2004). Manure characteristics:. MWPS‐18 Section 1. MWPS, https://www.canr.msu.edu/uploads/files/ManureCharacteristicsMWPS‐18_1.pdf

[jeq270292-bib-0025] Marvinney, E. , & Kendall, A. (2021). A scalable and spatiotemporally resolved agricultural life cycle assessment of California almonds. The International Journal of Life Cycle Assessment, 26, 1123–1145.

[jeq270292-bib-0026] Matthews, W. A. , & Sumner, D. A. (2019). Contributions of the California dairy industry to the California economy in 2018 . University of California Agricultural Issues Center.

[jeq270292-bib-0027] Meyer, D. , Heguy, J. , Karle, B. , & Robinson, P. (2019). Characterize physical and chemical properties of manure in California dairy systems to improve greenhouse gas emissions (Final report: Contract No. 16RD002). California Air Resources Board.

[jeq270292-bib-0028] Meyer, D. , Price, P. L. , Rossow, H. A. , Silva‐del‐Rio, N. , Karle, B. M. , Robinson, P. H. , DePeters, E. J. , & Fadel, J. G. (2011). Survey of dairy housing and manure management practices in California. Journal of Dairy Science, 94(9), 4744–4750. 10.3168/jds.2010-3761 21854949

[jeq270292-bib-0029] Miito, G. J. , Ndegwa, P. , Alege, F. P. , Coulibaly, S. S. , Davis, R. , & Harrison, J. (2021). A vermifilter system for reducing nutrients and organic‐strength of dairy wastewater. Environmental Technology & Innovation, 23, 101648. 10.1016/j.eti.2021.101648

[jeq270292-bib-0030] Miller, C. M. F. , Price, P. L. , & Meyer, D. (2017). Mass balance analyses of nutrients on California dairies to evaluate data quality for regulatory review. Science of the Total Environment, 579, 37–46. 10.1016/j.scitotenv.2016.10.092 27871748

[jeq270292-bib-0031] Newtrient . (2025). Environmental solutions for dairy . Newtrient. https://www.newtrient.com/

[jeq270292-bib-0032] Newtrient . (2026). Newtrient solutions catalogue . https://www.newtrient.com/newtrient‐solutions‐catalog/

[jeq270292-bib-0033] Njuki, E. (2022). Sources, trends, and drivers of U.S. dairy productivity and efficiency (ERR‐305). U.S. Department of Agriculture, Economic Research Service.

[jeq270292-bib-0035] Oenema, O. , Kros, H. , & de Vries, W. (2003). Approaches and uncertainties in nutrient budgets: Implications for nutrient management and environmental policies. European Journal of Agronomy, 20, 3–16.

[jeq270292-bib-0051] Overheid.nl . (2025). Fertilizer Act, https://www.wetten.overheid.nl/BWBR0004054/2025‐12‐09

[jeq270292-bib-0036] Parsons, T. , & Harter, T. (2018). Fate of nitrogen on California central valley dairies as measured by regulatory reporting [Master's thesis, University of California]. ProQuest.

[jeq270292-bib-0038] PE International . (2019). GaBi ts 6.0: System software and databases for life cycle engineering. PE International.

[jeq270292-bib-0039] Pettygrove, G. S. , Heinrich, A. L. , & Eagle, A. J. (2009). Dairy manure nutrient content and forms . (Bulletin #134369). UCCE. http://manauremanagement.ucdavis.edu/files/134369.pdf

[jeq270292-bib-0040] Porterfield, K. K. , Faulkner, J. , & Roy, E. D. (2020). Nutrient recovery from anaerobically digested dairy manure using dissolved air flotation (DAF). ACS Sustainable Chemistry & Engineering, 8(4), 1964–1970. 10.1021/acssuschemeng.9b06419

[jeq270292-bib-0041] Powell, J. M. , & Wu, Z. (1999). Nitrogen‐15 labeling of dairy feces and urine for nutrient cycling studies. Agronomy Journal, 91, 814–818.

[jeq270292-bib-0052] Sommer, S. G. , & Knudsen, L. (2021). Impact of Danish livestock and manure management regulations on nitrogen pollution, crop production and economy. Frontiers in Sustainability, 2. 10.3389/frsus.2021.6582312

[jeq270292-bib-0054] Splinter, M. A. B. S. , & Peerlings, J. H. M. (2023). Examining the trade‐offs between agri‐environmental and manure policies in Dutch dairy farming. NJAS: Impact in Agricultural and Life Sciences, 95(1). 10.1080/27685241.2023.2194261

[jeq270292-bib-0042] Stacey, N. , Hills, K. , & Yorgey, G. (2021). Estimating and comparing cropland nitrogen need with dairy farm nutrient recovery: A case study in Whatcom County, WA. Renewable Agriculture and Food Systems, 36(2), 130–137. 10.1017/S1742170520000198

[jeq270292-bib-0043] UC Davis Agricultural and Resource Economics (UCD ARE) . (2021). Current cost and return studies . UCD ARE. https://coststudies.ucdavis.edu/current/commodities

[jeq270292-bib-0046] United States Environmental Protection Agency (USEPA) . (2017). Tool for reduction and assessment of chemicals and other environmental impacts (TRACI) . USEPA. https://www.epa.gov/chemical‐research/tool‐reduction‐and‐assessment‐chemicals‐and‐other‐environmental‐impacts‐traci

[jeq270292-bib-0056] USDA_NASS . (2020). Cropland data layer, https://www.nass.usda.gov/ResearchandScience/Cropland?SARS1a.php

[jeq270292-bib-0047] Vanotti, M. B. , García‐González, M. C. , Szögi, A. A. , Harrison, J. H. , Smith, W. B. , & Moral, R. (2020). Removing and recovering nitrogen and phosphorus from animal manure. In Animal manure: Production, characteristics, environmental concerns, and management (pp. 275–321). Wiley.

[jeq270292-bib-0048] Yin, Y. , Guo, S. , Xu, Q. , Liu, J. , Wang, H. , Zhuang, Y. , Wang, Z. , Hua, L. , Liu, H. , & Zhai, L. (2025). Co‐benefits for cropland yield, nitrogen emissions, and climate impact through multi‐objective optimization agricultural manure solutions. Nature Communications, 16(1), 6415. 10.1038/s41467-025-61885-w PMC12254328 40645999

