# Supplementary material for: Manure nutrient recovery, removal, and reuse on California dairies
Source: J Environ Qual. 2026 Oct 22;55(5):e70292. doi: 10.1002/jeq2.70292 (PMC13691170; doi:10.1002/jeq2.70292)
Supplement: Supplementary file 1 [file JEQ2-55-0-s001.docx]

**Supplemental Information**

**Manure Nutrient Recovery, Removal and Reuse on California Dairies**

Stephen R. Kaffka, Department of Plant Sciences, University of California, Davis, One Shields Ave, Davis CA 95616

Cole Smith, Department of Land, Air, and Water Resources, University of California, Davis, One Shields Ave, Davis CA 95616

Elias Marvinney, Department of Civil and Environmental Engineering; University of California, Davis, One Shields Ave, Davis CA 95616

**SI 1. Dairy farm characteristics and stocking rates**

**a**

**b**

**Figure SI 1.** Aerial images of a typical open lot dairy (a) and large free-stall dairy (b). On the open lot dairy, producing cows and other stock are kept in pens and manure deposited is periodically scrapped and removed to storage piles or directly field applied between crops. (b) The majority of producing cows are housed in the free stall barn where manure is flushed with water or scraped into collection pits and stored in lagoons. Lagoon water is used for irrigation. Young stock and dry cows are kept in open lots, indicating the mix of manure management systems commonly used. These differing management systems affect the management of N surpluses in manure and the potential for adoption of new manure nutrient management technologies. Photos courtesy of Dairy Cares (<https://www.dairycares.com/> ).

| **Table SI 1. Estimated surplus N in manure based on stocking rates, collection system, estimated crop uptake, AR = 1.4, and accounting for manure export of SM** | | | | | |
| --- | --- | --- | --- | --- | --- |
| **Cows/ha** | **Manure N** | **Forage crops/yr** | | | |
|  | **(kg/ha/yr/coweq)** | **2** | **3** | **2** | **3** |
|  | **(excreted)** | **Free stall dairy** | | **Open Lot Dairy** | |
| **2.5** | **493** | **-305** | **-465** | **-331** | **-491** |
| **4.9** | **988** | **-39** | **-199** | **-90** | **-250** |
| **7.4** | **1482** | **227** | **67** | **150** | **-10** |
| **9.9** | **1976** | **492** | **332** | **389** | **229** |
| **12.4** | **2470** | **758** | **598** | **629** | **469** |
| **14.8** | **2964** | **1023** | **863** | **869** | **709** |
| **17.3** | **3458** | **1289** | **1129** | **1109** | **949** |
| **19.8** | **3952** | **1555** | **1395** | **1349** | **1189** |
| **22.2** | **4446** | **1820** | **1660** | **1589** | **1429** |
| **24.7** | **4940** | **2086** | **1926** | **1828** | **1668** |
| ***Notes***: SR are rounded to whole numbers. Cows + replacements; Manure per cow equivalent (lactating cows + replacements) = 200 kg N/yr | | | | | |
| 2 crops/yr: cereal silage and corn silage, N uptake = 450 kg N/ha | | | | | |
| 3 crops/yr: cereal silage, corn silage, Sudan grass hay; N uptake = 560 kg N /ha | | | | | |
| AR = 1.4 times manure N available for application | | | | | |
| N application: 2 crop system =630 kg N/ha-56 kg N as fertilizer applied to corn silage | | | | | |
| N application: 3 crop system =784 kg N/ha/yr - 56 kg N fertilizer applied to corn silage | | | | | |
| Free stall: 60% on concrete (70% recovery of N); 40 % in corrals (63% recovery of N) | | | | | |
| Open lot: 35% on concrete (60 % recovery), 65 % in corrals. | | | | | |

| **Table SI 2. Surplus N on Priority one NMZ dairies reporting a stocking rate ≥ 10 mature cows per ha.** | | | | | | |
| --- | --- | --- | --- | --- | --- | --- |
|  | **Chowchilla** | **Modesto** | **Turlock** | **Kaweah** | **Kings** | **Tule** |
| **# of farms** | **10** | **20** | **81** | **44** | **43** | **44** |
| **% of farms** | **32** | **52** | **58** | **63** | **50** | **55** |
| **ha receiving manure** | **672** | **1713** | **5532** | **4192** | **4386** | **6597** |
| **Lagoon manure N (Mg/yr)** | **299** | **635** | **3111** | **1950** | **2549** | **2703** |
| **Range (FS:OL)** | 399-145 | 862-317 | 3918-1442 | 2585-830 | 2521-871 | 3682-1297 |
| **Solid manure N (Mg/yr)** | **245** | **553** | **2857** | **889** | **1642** | **2340** |
| **Range (FS:OL)** | 195-336 | 608-707 | 2263-4753 | 1977-3211 | 1424-2041 | 2023-3066 |
| Notes: FS: Freestall; OL: Open Lot. | | | | | | |
| Averages are the mean of 2 and 3 crop systems (N uptake*AR =650 kg N / ha applied) | | | | | | |
| Lagoon manure (LM) is from FS and OL farms combined. Similarly for solid manure (SM) | | | | | | |

| **Table SI 3. Numbers and percent of dairies by different stocking rates and NMZ** | | | | | | | | | | | | |
| --- | --- | --- | --- | --- | --- | --- | --- | --- | --- | --- | --- | --- |
|  | **Chowchilla** | | **Turlock** | | **Modesto** | | **Kaweah** | | **Kings** | | **Tule** | |
| ***Stocking rate***  ***(Cows ha^-1^)*** | **Dairies** | | **Dairies** | | **Dairies** | | **Dairies** | | **Dairies** | | **Dairies** | |
|  | **(n)** | **%** | **(n)** | **%** | **(n)** | **%** | **(n)** | **%** | **(n)** | **%** | **(n)** | **%** |
| *2.5* | 1 | 3 | 7 | *4* | 0 | 0 | 9 | 9 | 12 | 11 | 2 | 2 |
| *5* | 12 | 39 | 21 | *13* | 8 | 18 | 12 | 11 | 19 | 18 | 11 | 12 |
| *7.5* | 7 | 23 | 32 | *20* | 11 | 24 | 18 | 17 | 22 | 21 | 29 | 31 |
| *10 (8.8 to 11)* | 1 | 3 | 27 | *17* | 8 | 18 | 25 | 24 | 16 | 15 | 8 | 9 |
| *12.5* | 2 | 6 | 13 | *8* | 5 | 11 | 8 | 8 | 11 | 10 | 19 | 20 |
| *15* | 3 | 10 | 11 | *7* | 5 | 11 | 10 | 10 | 6 | 6 | 8 | 9 |
| *17.5* | 1 | 3 | 13 | *8* | 1 | 2 | 6 | 6 | 5 | 5 | 4 | 4 |
| *20* | 1 | 3 | 8 | *5* | 3 | 7 | 4 | 4 | 3 | 3 | 2 | 2 |
| *22.5* | 3 | 10 | 4 | *3* | 2 | 4 | 4 | 4 | 5 | 5 | 4 | 4 |
| *25* | 0 | 0 | 22 | *14* | 2 | 4 | 9 | 9 | 6 | 6 | 6 | 6 |
| *SUM* | 31 |  | 158 |  | 45 |  | 105 |  | 105 |  | 93 |  |
| *Dairies with a SR of cows/ha* ≥ 10 | 10 | *32* | 98 | 62 | 26 | *58* | 66 | *63* | 52 | *50* | 51 | 55 |

| **Table SI 4a. Turlock NMZ; surplus N by stocking rate for farms with ≥ 10 cows per ha** | | | | | | | | | | | |
| --- | --- | --- | --- | --- | --- | --- | --- | --- | --- | --- | --- |
| **SR** | **Dairies** | **Ha** | **Cows** | **SURPLUS N (Mg N yr^-1^)** | | | | **Contribution %** | | | |
|  |  |  |  | **Free stall** | | **Open Lot** | |  |  |  |  |
|  |  | **Sum** | **Sum** | **Crops per year** | | | | **Farms** | **Acres** | **Cows** | **N** |
| **(Cows ha^-1^)** | **(#)** | **(ha)** | **(cows)** | **2** | **3** | **2** | **3** | **(%)** | **(%)** | **(%)** | **(%)** |
| *10 to 12* | 17 | 2080 | 22250 | 1241 | 911 | 1005 | 681 | 21 | 38 | 24 | 16 |
| *12 to 15* | 12 | 763 | 10477 | 742 | 620 | 627 | 508 | 15 | 14 | 11 | 10 |
| *15 to 17* | 14 | 985 | 15859 | 1182 | 1024 | 1012 | 858 | 17 | 18 | 17 | 16 |
| *17 to 20* | 7 | 513 | 9363 | 708 | 626 | 610 | 531 | 9 | 9 | 10 | 10 |
| *20 to 22* | 5 | 263 | 5378 | 422 | 379 | 366 | 325 | 6 | 5 | 6 | 6 |
| *22 to 25* | 4 | 110 | 2533 | 207 | 189 | 180 | 163 | 5 | 2 | 3 | 3 |
| *25 to 27* | 5 | 139 | 3567 | 297 | 272 | 259 | 238 | 6 | 3 | 4 | 4 |
| *27 to 30* | 6 | 262 | 7423 | 665 | 622 | 585 | 543 | 7 | 5 | 8 | 10 |
| *30 or more* | 12 | 416 | 17816 | 1670 | 1599 | 1468 | 1403 | 15 | 8 | 19 | 25 |
| *Total* | 82 | 5533 | 94666 | 7132 | 6242 | 6112 | 5248 | 100 | 100 | 100 | 100 |

| **Table SI 4b. Tule NMZ; surplus N by stocking rate for farms with ≥ 10 cows per ha** | | | | | | | | | | | |
| --- | --- | --- | --- | --- | --- | --- | --- | --- | --- | --- | --- |
| **SR** | **Dairies** | **Land available for manure** | **Cows** | **SURPLUS N (Mg N yr^-1^)** | | | | **Contribution %** | | | |
|  |  |  |  | **Free stall** | | **Open Lot** | |  |  |  |  |
|  |  |  |  | **Crops per year** | | | | **Farms** | **Acres** | **Cows** | **N** |
| **(Cow ha^-1^)** | **(#)** | **(ha)** | **(cows)** | **2** | **3** | **2** | **3** | **(%)** | **(%)** | **(%)** | **(%)** |
| *10 to 12* | 18 | 3744 | 40987 | 2398 | 1811 | 1965 | 1379 | 41 | 57 | 44 | 33 |
| *12 to 15* | 10 | 1566 | 20584 | 1390 | 1143 | 1171 | 925 | 23 | 24 | 22 | 20 |
| *15 to 17* | 4 | 559 | 8894 | 632 | 544 | 541 | 454 | 9 | 8 | 10 | 9 |
| *17 to 20* |  | 0 |  |  |  |  |  |  |  |  | 0 |
| *20 to 22* | 2 | 165 | 3263 | 258 | 233 | 225 | 199 | 5 | 2 | 3 | 4 |
| *22 to 25* | 4 | 251 | 477 | 472 | 374 | 331 | 298 | 9 | 4 | 1 | 6 |
| *25 to 27* | 3 | 347 | 9255 | 820 | 766 | 722 | 668 |  | 5 | 10 | 13 |
| *27 to 30* |  | 0 |  |  |  |  |  |  |  |  |  |
| *30 or more* | 3 | 222 | 9990 | 963 | 928 | 858 | 824 | 7 | 3 | 11 | 15 |
| *Total* | 44 | 6853 | 93450 | 6933 | 5798 | 5813 | 4745 | 100 | 100 | 100 | 100 |
|  |  |  |  |  |  |  |  |  |  |  |  |
|  |  |  |  |  |  |  |  |  |  |  |  |

| **Table SI 5a. Turlock NMZ; surplus N by farm size (available ha) for farms with ≥ 10 cows per ha** | | | | | | | | | | | | |
| --- | --- | --- | --- | --- | --- | --- | --- | --- | --- | --- | --- | --- |
| **Dairie size by category** | **Dairies** | **Available land for manure** | **SR** | **Cows** | **SURPLUS N**  **(Mg N yr^-1^)** | | | | **Contribution %** | | | |
|  |  |  |  |  | **Free stall** | | **Open Lot** | |  |  |  |  |
|  |  |  |  |  | **Crops per year** | | | |  |  |  |  |
|  |  | **Sum** | **Average** | **Sum** | **2** | **3** | **2** | **3** | **Farms** | **ha** | **Cows** | **N** |
| **(ha)** | **(#)** | **(ha)** | **(cows/ha) (range)** | **(cows)** |  | | | | **(%)** | **(%)** | **(%)** | **(%)** |
| *<40* | 37 | 895 | 23.5 (12.4 - 62) | 20341 | 1685 | 1545 | 1475 | 1334 | 46 | 16 | 21 | 24 |
| *40-80* | 21 | 1256 | 23.7 (9.9 - 52) | 29894 | 2521 | 2325 | 2198 | 2001 | 26 | 23 | 32 | 36 |
| *80-120* | 12 | 1181 | 12.8 (9.9 - 20) | 15372 | 986 | 801 | 827 | 642 | 15 | 21 | 16 | 13 |
| *120-160* | 3 | 465 | 16.5 | 7696 | 567 | 493 | 487 | 414 | 4 | 8 | 8 | 8 |
| *160-200* | 3 | 368 | 15.8 | 5783 | 430 | 372 | 368 | 311 | 4 | 7 | 6 | 6 |
| *200-240* | 2 | 444 | 13.3 | 5810 | 393 | 323 | 331 | 261 | 2 | 8 | 6 | 5 |
| *240-280* | 0 |  |  |  |  |  |  |  |  |  |  |  |
| *280-320* | 2 | 583 | 11.1 | 6430 | 375 | 284 | 307 | 217 | 2 | 11 | 7 | 5 |
| *320-360* | 1 | 341 | 9.9 | 3340 | 174 | 121 | 140 | 85 | 1 | 6 | 4 | 2 |
| *Total* | 81 | 5533 |  | 94666 | 7132 | 6263 | 6132 | 5265 | 100 | 100 | 100 | 100 |
| AVERAGE N SURPLUS | | | | | 6700 | | 5700 | |  | | | |
| SURPLUS Weighted average (67% FS: 33% OL) = 6370 Mg N yr^-1^ | | | | | | | | | | | | |
| SURPLUS Weighted average (90% FS: 10% OL) = 6600 Mg N yr^-1^ | | | | | | | | | | | | |

| **Table SI 5b. Tule NMZ; surplus N by farm size (available acres) for farms with ≥ 10 cows per ha** | | | | | | | | | | | | |
| --- | --- | --- | --- | --- | --- | --- | --- | --- | --- | --- | --- | --- |
| **Dairy size by category** | **Dairies** | **Available land for manure** | **SR** | **Cows** | **SURPLUS N (Mg N yr^-1^)** | | | | **Contribution %** | | | |
|  |  |  |  |  | **Free stall** | | **Open Lot** | |  |  |  |  |
|  |  |  |  |  | **Crops per year** | | | |  |  |  |  |
|  |  | **Sum** | **Average** | **Sum** | **2** | **3** | **2** | **3** | **Farms** | **ha** | **Cows** | **N** |
| **(ha)** | **(#)** | **(ha)** | **(cows/ha)** | **(cows)** |  | | | | **(%)** | **(%)** | **(%)** | **(%)** |
| *<40* | 4 | 106 | 41 | 4470 | 422 | 405 | 375 | 358 | 9 | 2 | 5 | 7 |
| *40-80* | 6 | 381 | 17 | 6296 | 469 | 409 | 404 | 344 | 14 | 6 | 6 | 7 |
| *80-120* | 6 | 621 | 18 | 10519 | 771 | 673 | 663 | 566 | 14 | 9 | 11 | 11 |
| *120-160* | 10 | 1341 | 19 | 25539 | 2034 | 1824 | 1766 | 1556 | 23 | 20 | 26 | 31 |
| *160-200* | 5 | 935 | 13 | 12238 | 774 | 627 | 649 | 502 | 11 | 14 | 12 | 11 |
| *200-240* | 4 | 910 | 12 | 11077 | 705 | 561 | 588 | 445 | 9 | 13 | 11 | 10 |
| *240-280* | 6 | 1590 | 12 | 17941 | 1085 | 835 | 894 | 654 | 14 | 23 | 18 | 15 |
| *280-320* | 2 | 624 | 11 | 7095 | 405 | 307 | 333 | 235 | 5 | 9 | 7 | 5 |
| *320-360* | 1 | 346 | 13 | 3850 | 268 | 214 | 224 | 170 | 2 | 5 | 4 | 4 |
| *Total* | 44 | 6853 |  | 99025 | 6933 | 5856 | 5896 | 4830 | 100 | 100 | 100 | 100 |
| AVERAGE N SURPLUS | | | | | 6400 | | 5360 | |  |  |  |  |
| SURPLUS Weighted average (67% FS: 33% OL) = 6060 Mg N yr^-1^ | | | | | | | | | | | | |
| SURPLUS Weighted average (90% FS: 10% OL) = 6300 Mg N yr^-1^ | | | | | | | | | | | | |

**SI 2: Global warming potential analysis**

| **Table SI 6. Life cycle greenhouse gas impacts of various dairy waste management/ nutrient recovery processes generated using the SPARCS-LCA model (Marvinney and Kendall 2021).** | | | | |
| --- | --- | --- | --- | --- |
| **Process** | **GWP₂₀**  **(kg CO_2_eq kg ^-1^ input DM )** | **GWP₁₀₀**  **(kg CO_2_eq kg^-1^ input DM)** | **% lagoon baseline (GWP₂₀)** | **% lagoon baseline (GWP₁₀₀)** |
| *Compost/ Windrow** | 3.02 | 1.01 | -- | -- |
| *DAF*** | -0.002 | -0.003 | -- | -- |
| *MVR (3.7% influent solids)*** | 2.20 | 0.91 | -- | -- |
| *MVR (0.6% influent solids)*** | 2.89 | 1.48 | -- | -- |
| *VERM*** | 0.10 | 0.02 | -- | -- |
| *Lagoon^†^* | **8.63** | **3.09** | **100.00%** | **100.00%** |
| *DAF + Lagoon* | 8.63 | 3.09 | 99.98% | 99.91% |
| *MVR (3.7% influent solids) + Lagoon* | 10.83 | 4.01 | 125.49% | 129.43% |
| *MVR (0.6% influent solids) + Lagoon* | 11.53 | 4.58 | 133.53% | 147.86% |
| *VERM + Lagoon* | 1.56 | 0.57 | 101.19% | 100.59% |
| *Anaerobic Digestion* | 0.31 | 0.14 | 3.60% | 4.51% |
| *DAF + Anaerobic Digestion* | 0.31 | 0.14 | 3.58% | 4.42% |
| *MVR (3.7% influent solids) + Anaerobic Digestion* | 2.51 | 1.05 | 29.09% | 33.94% |
| *MVR (0.6% influent solids) + Anaerobic Digestion* | 3.20 | 1.62 | 37.08% | 52.24% |
| *VERM + Anaerobic Digestion* | 0.22 | 0.10 | 2.49% | 3.12% |
| Notes:  *Solid waste stream process - not directly comparable with lagoon liquid waste stream baseline.  **Assumed to be used only in combination with lagoon storage or anaerobic digestion - not directly comparable with lagoon baseline.  ^†^Baseline, “business-as-usual” scenario. | | | | |

**SI 3: Evaluation of Selected Nutrient Recovery Systems**

Analysis of the performance of selected dairy nutrient recovery and reuse systems is based on limited amounts of published data to date where available, and in this analysis, also includes information provided by companies supplying the technologies discussed. Greater detail is provided in Kaffka et al., (2022).

The DAF Nutrient Recovery System analyzed treats liquid or slurry manure using a dissolved air flotation system (DAF) to recover the majority of P and smaller amounts of N and potassium (K) in the separated fine solids. The system is modular and typically consists of course solids removal followed by a standard flocculant (typically polyacrylamide) and DAF system for fine solids removal. The flocs or coagulated fine particles float to the surface in the DAF vessel assisted by small air bubbles. The floating sludge-like material is skimmed and passed through a disc press dewatering the material to about 20% dry matter (sometimes called "fine solids cake"). The combined effluent from the DAF and the disc press, with relatively low solids content, flows to storage for reuse as flush water and eventual irrigation (Figure 2.2).

Figure SI 2. Typical layout of the DAF system at a flush dairy

DAF was developed in Sweden in 1965 for use in clarifying drinking water (Crossley & Valade, 2006) and is used primarily in the drinking water and pulp and paper industries. Because of its ability remove fine solids and partition nutrients, more than 20 DAF systems have been installed on dairies in the U.S. (Stacey et al., 2021).

**Nutrient Recovery.** DAF systems can recover up to 90% of the P and smaller amounts of N and K. Roughly half of the N (organic N and nearly all the ammoniac N) and more than 80%+ of the K remains in the liquid effluent.

**Vermilfiltration**

Vermifiltration (VERM) wastewater biotreatment system consisting of a layer of wood chips or shavings inoculated with earthworms in the upper portion. Beneath the woodchips is a porous layer of gravel or river cobble. The bottom of the system is a catchment or drainage basin (Figure 2.3). Wastewater is applied to the top surface and trickles down through the media where it is degraded by symbiotic activities of earthworms and microorganisms (Zhou et al., 2010). The VERM system is primarily an aerobic bioreactor, maintained by tunneling activities of the earthworms, with anaerobic conditions inside the worm gut and small non-aerated pockets in the media (Lai et al., 2018). Periodically (i.e., every twelve to eighteen months), the organic media along with accumulated worm castings and retained influent solids are removed and can used as fertilizer or compost. Vermifiltration was added to CDFA’s AMMP program in 2021.

Figure SI 3. Schematic of the BIDA vermifilter.

With vermifiltration, ammonia N is reduced via biological nitrification/denitrification producing mainly N gas (N_2_) with a trace amounts of N_2_O (Frear et al., 2018, Lai et al., 2019). A portion of the influent P and K remains in the media in captured solids, worm castings and adsorbed onto the wood chips. Solids and volatile solids in the effluent are reduced by biological activity and/or trapped in the media.

**Nutrient and Solids Reduction.** For systems treating dairy manure, total N in the effluent is reduced 40%-90% compared to that in the influent. Phosphorous (P) and potassium (K) reduction range from 20%-80% and 0-25%, respectively. Total solids reduction is 30%-80% while volatile solids are reduced 80%-90%. This fairly large range in nutrient and solids reduction is likely due to differences in manure management and flush rates, placement of the vermifilter within the manure management system (before or after a wastewater storage lagoon), amount of course solids removal prior to the vermifilter, scale (pilot or full flow treatment), climate, etc. among the systems in the cited literature.

**System Layout.** For treating flush dairy manure, coarse solids need to be removed from the flush stream prior to entering the vermifilter. The liquid effluent from the vermifilter would be sent to a storage lagoon for reuse as flush water and/or eventual irrigation. The preferred placement of a vermifilter at a flush dairy is shown in Figure SI 4. Some installations put the vermifilter after the primary storage lagoon and upstream of a second (or effluent) lagoon.

Figure SI 4. Schematic showing manure flow at a flush dairy with a vermifilter.

A vermifilter for a dairy would require about 0.9 – 1.2 ft^2^ surface area per gallon-per-day (GPD) of flow with less than 3% total suspended solids in the inlet (BioFiltro 2022). Therefore, land area required for a vermifilter would vary from about 0.6 acre for a 500 cow flush dairy to 12.5 acres for a 10,000 cow dairy.

**Methane Emissions.** Lai et al. 2018 measured methane emissions of about 0.8 kg/day from a BioFiltro vermifilter at a California dairy. The vermifilter was 539 m^2^ in size and designed to process manure from about 150 adult cows (after coarse solids separation). Assuming 60% of total manure is collected in the flush system, and 30% solids removal in the separator, the BioFiltro unit has a methane emission factor of approximately~0.0019 kg CH_4_/kg VS which is about 98% lower than the emissions factor for anaerobic lagoons. Dore et al., 2022 measured methane emissions at the same vermifilter and found the emission factor was approximately 99% lower for the vermifilter compared to an anaerobic lagoon.

Overall system methane emissions for a vermifilter that discharges into a storage lagoon (anaerobic lagoon), see Figure SI 4, is about 0.026 kg CH4 per kg of VS that enters the vermifilter. This is about 78% less methane emitted than the system without the vermifilter (i.e., anaerobic lagoon only).

**Mechanical Vapor Recompression**

The MVR system separates solids from a liquid or slurry input producing an aqueous ammonia stream (~80 g NH4 /L^-1^), relatively clean water (~30 mg NH4 L^-1^) and low moisture solids. The solid and liquid fractions of the input stream are separated by thermal evaporation and the resulting vapor is sent to a compressor, where it undergoes Mechanical Vapor Recompression (MVR). The latent heat in the compressed vapor is then used as the heat source for the evaporation process (Figure 2.5). The compressed vapor condenses to a liquid after transferring its latent heat to the dryer. The liquid flows through the ammonia distillation system where an aqueous ammonia distillate stream is produced separate from the main condensate flow. MVR is used widely in applications that require concentrating or thickening a liquid (i.e., food, beverage, pharmaceutical, chemical industries, and zero liquid discharge wastewater treatment). Energy inputs include electricity, mainly for the compressor and, after initial start-up, a relatively small amount of natural gas to make up for equipment heat loss.

Figure SI 5. MVR Schematic

The MVR system can treat pumpable slurries with solids content less than about 10%. For application at dairies, the system is best suited for those that use scrape or vacuum manure collection and/or have effluent from a high solids digester (e.g., plug flow or continuously stirred tank design).

**Nutrient Distribution**

Ninety percent or more of the inlet ammonia is recovered in the aqueous ammonia distillate output stream. This could be used as a pathogen-free ammonium liquid fertilizer with about 10% N concentration and a pH of 7. The dried solids output retains essentially all of the inlet organic N as well as all of the inlet P and K. This could be used as weed and pathogen-free solid fertilizer. Finally, the relatively clean water (condensate) output could be recycled at the point of use. The solids output product has been certified “organic” by the Organic Materials Review Institute (OMRI).

**Energy Requirement**

The MVR system is treating effluent from a plug-flow digester at a 3500 cow dairy in the Midwest that utilizes vacuum manure collection. It processes 90 gallons per minute at approximately 3.7% solids. The energy to operate the system is about 80 MMBtu/day (84,400 MJ/day) of natural gas and 14 MWh/day of electricity (Sedron 2022). This is about 1/8^th^ of the energy needed for a dryer with no latent heat recovery (i.e., without MVR).^[1]^ On a per-kg solids basis, the energy required to separate solids climbs steeply as the influent solids content decreases.

**SI 4. Nitrogen Availability of by-products**

Solid by-products from the nutrient recovery technologies were evaluated for potentially mineralizable N (PMN) following the general approach described by Curtin and Campbell, 2006). Amendment derived N was calculated by subtracting the inorganic N values of amended soils from those of the unamended, in addition to the initial inorganic N present at time zero:

N_min_ =ΔN_t_ −ΔN_t_=0

ΔN_t_ =(N_min,t_ )_trt_ −(N_min,t_ )_con_

ΔN_t=0_ =(N_min,t=0_ )_trt_ −(N_min,t=0_ )_con_

Total added organic N was calculated as the difference between total applied N and initial inorganic N:

N_org_=500−ΔN_t=0_

The percentage of organic N mineralized at time *t* was then calculated as:

%N_org_=(N_min/_N_org_)×100%

Results from these analyses were used to estimate the nutrient replacement value as well as for techno-economic assessment (TEA) and life cycle assessment (LCA) modeling. Further discussion can be found in Kaffka et al., 2022.

Figure SI 6. Total mineralized N for the 5 amendments Compost (COM) Vermicompost (VER), Livestock Water Recycling product (LWR), Dissolved Aerated Flotation (DAF), Solids from mechanical vapor recompression (MVR) shown as a percentage of added organic N incubated at 3 temperatures (10°C, 20°C, 30°C) in two soil textures (Clay and Sand) for 28 days. The LWR technology (<https://www.livestockwaterrecycling.com> ) is a post-collection membrane based solid separator that produces an N-rich solid material like DAF and included in the N mineralization for comparison to similar products.

**SI 5. Life Cycle Assessment**

The three recovery technology scenarios are represented graphically to compare the framework for calculation of environmental impacts measured per unit mass of manure dry matter input. They illustrate different types of manure storage on dairy farms and include manure flows before and after treatment by each selected technology and subsequent distribution and potential use pathways

**Figure SI 7**. Life cycle assessment system diagrams. Dashed line represents the analytic system boundary containing those processes evaluated for environmental performance, expanded to include displacement of fertilizer production. Each nutrient recovery technology is treated as a black box, in the sense that individual process components and energy demand are aggregated based on results of techno-economic analysis as described above. A) Dissolved Air Flotation system. B) Mechanical Vapor Recompression (MVR) system. C) Vermifiltration (VERM) system. D) Generalized alternative nutrient recovery system diagram.

**SI-References**

Angermann, T. (2019). Memo: "Site visit to Windy Ridge Dairy and Midwestern BioAg Fertilizer Plant, Fair Oaks, Indiana". Luhdorff & Scalmanini Consulting Engineers.

BioFiltro (2022). Personal communication.

Canter, T., Lim, T. T., & Zulovich, J. (2021). Nutrient recovery system for dairy farms : dissolved air flotation and multi-disk press (University of Missouri Extension No. eq303). Retrieved from <https://mospace.umsystem.edu/xmlui/bitstream/handle/10355/85108/EQ0303.pdf?sequence=1&isAllowed=y>

Crossley, I. A., & Valade, M. T. (2006). A review of the technological developments of dissolved air flotation. *Journal of Water Supply: Research and Technology - AQUA*, *55*(7–8), 479–491. <https://doi.org/10.2166/aqua.2006.057>

Curtin, D. and C.A. Campbell. 2006. Mineralizable nitrogen: In: M.R. Carter and E.G. Gregorich (eds.). Soil sampling and methods of analysis. 2^nd^ ed. CRC Press, Boca Raton, FL.

Dore, S., Deverel, S., Iacobelli, A., & Sjögren, M. (2019). White Paper: The BioFiltro BIDA Wastewater Treatment System. *Hydro Focus / BioFiltro*.

Dore S, Deverel SJ, Christen N (2022). A vermifiltration system for low methane emissions and high nutrient removal at a California dairy. Bioresource Technology Reports. 2022 Jun 1;18:101044. Frear, C., Ma, J., & Yorgey, G. (2018). Approaches to Nutrient Recovery from Dairy Manure. Washington State University Extension Publication EM112E.

Jensen, J., C. Frear, J. Ma, C. Kruger, R. Hummel, and G. Yorgey. 2016. Digested Fiber Solids—Methods for Adding Value. WSU Extension Factsheet FS35E, Pullman, WA.

Lai, E., Hess, M., & Mitloehner, F. M. (2018). Profiling of the microbiome associated with nitrogen removal during vermifiltration of wastewater from a commercial dairy. Frontiers in Microbiology, 9(AUG), 1–13. <https://doi.org/10.3389/fmicb.2018.01964>

Miito, G. J. (2021). *Vermifiltration as a Low-Cost Nutrient Recovery Technology for Managing Dairy Wastewater*. PhD Dissertation. Department of Biological Systems Engineering, Washington State University. Retrieved from <https://rex.libraries.wsu.edu/esploro/outputs/doctoral/VERMIFILTRATION-AS-A-LOW-COST-TECHNOLOGY-FOR/99900652103801842>

Miito, G. J., Ndegwa, P., Alege, F. P., Coulibaly, S. S., Davis, R., & Harrison, J. (2021). A vermifilter system for reducing nutrients and organic-strength of dairy wastewater. Environmental Technology and Innovation, 23, 101648. <https://doi.org/10.1016/j.eti.2021.101648>

Newtrient (2022). Company Description: Trident TNZ LLC Nutrient Recovery Process. Accessed March 2022: [https://www.newtrient.com/catalog/trident-processes-llc-trident-nutrient-recovery/#](https://www.newtrient.com/catalog/trident-processes-llc-trident-nutrient-recovery/)

Pasha, M. F. K., Yeasmin, D., Zoldoske, D., Kc, B., & Hernandez, J. (2018). Performance of an Earthworm-Based Biological Wastewater-Treatment Plant for a Dairy Farm: Case Study. Journal of Environmental Engineering, 144(1), 04017086. <https://doi.org/10.1061/(asce)ee.1943-7870.0001290>

Porterfield, K. K., Faulkner, J., & Roy, E. D. (2020). Nutrient Recovery from Anaerobically Digested Dairy Manure Using Dissolved Air Flotation (DAF). *ACS Sustainable Chemistry and Engineering*, *8*(4), 1964–1970. <https://doi.org/10.1021/acssuschemeng.9b06419>

Rotz, A., Stout, R. Leytem, A., Feyoreisen, G., Waldri, H., Thoma, G., Holly, M., Bjorneberg, D., Baker, J., Vadas, P. and Kleinman, P. (2021). Environmental assessment of United States dairy farms. J. Cleaner Prod. 315:128153.

Sedron (2022). Personal Communication

Rude, K., Yothers, C., Barzee, T.J., Kutney, S. Zhang, R., and Franz, A. (2022). Growth potential of microalgae on ammonia-rich anaerobic digester effluent for wastewater remediation. Algal Res. 62 102613

Stacey, N., Hills, K., & Yorgey, G. (2021). Estimating and comparing cropland nitrogen need with dairy farm nutrient recovery: A case study in Whatcom County, WA. *Renewable Agriculture and Food Systems*, *36*(2), 130–137. <https://doi.org/10.1017/S1742170520000198>

Timmermann, M. and Hoving , I.E. (2016). Purifying manure effluents with duckweed. Livestock Research. Wageningen UR. <https://library.wur.nl/WebQuery/wurpubs/livestock-reports/497717>

Zhao, L., Wang, Y., Yang, J., Xing, M., Li, X., Yi, D., et al. (2010). Earthworm-microorganism interactions: a strategy to stabilize domestic wastewater sludge. Water Res. 44, 2572–2582. doi: 0.1016/j.watres.2010.01.011

1. Energy to evaporate w/o MVR: 2.26 MJ/kg-water x 475,596 kg-water/day = 1,075,000 MJ/day
